# Post‐ERCP Outcomes in Cirrhotic Patients With Thrombocytopenia: A Propensity‐Matched Retrospective Comparative Analysis on TriNetX Health Research Database

**DOI:** 10.1002/jgh3.70418

**Published:** 2026-05-20

**Authors:** Umar Hayat, Azhar Hussain, Hassam Ali, Karan J. Yagnik, Pranav Patel, Ali A. Siddiqui, Sumant Inamdar, Kamran Qureshi, Harshit S. Khara, Bradley Confer, David L. Diehl

**Affiliations:** ^1^ Department of Internal Medicine Geisinger Wyoming Valley Medical Center Wilkes Barre Pennsylvania USA; ^2^ Department of Internal Medicine SUNY Upstate University Hospital Syracuse New York USA; ^3^ Department of Gastroenterology East Carolina University Greenville North Carolina USA; ^4^ Department of Internal Medicine Rutgers/Monmouth Medical Center Long Branch New Jersey USA; ^5^ Department of Gastroenterology Geisinger Medical Center Danville Pennsylvania USA; ^6^ Department of Gastroenterology Inova Fairfax Hospital, University of Virginia School of Medicine Falls Church Virginia USA; ^7^ Department of Gastroenterology University of Arkansas for Medical Sciences Little Rock Arkansas USA; ^8^ Department of Gastroenterology Saint Louis University St. Louis Missouri USA

**Keywords:** all‐cause mortality, choledocholithiasis, endoscopic retrograde cholangiopancreatography (ERCP), liver cirrhosis, post‐ERCP adverse events, thrombocytopenia

## Abstract

**Background:**

Patients with cirrhosis have an increased risk of developing complications secondary to thrombocytopenia. This study aims to investigate post‐ERCP outcomes in patients with liver cirrhosis and concomitant low platelet count who undergo endoscopic retrograde cholangiopancreatography (ERCP) for choledocholithiasis in a large, matched cohort with cirrhosis.

**Methods:**

A retrospective cohort study was conducted using data from the TriNetX research network. Patients aged 18 years or older with cirrhosis undergoing ERCP for choledocholithiasis were included. Cohorts were categorized by platelet count into mild or moderate thrombocytopenia groups, with 5097 patients in each cohort after propensity score matching, yielding a total of 10 194 patients. Univariate regression analysis was used to assess the primary outcome, namely, all‐cause mortality. Secondary outcomes included post‐ERCP bleeding, post‐ERCP pancreatitis, need for blood transfusion, and other adverse events such as sepsis and acute kidney injury (AKI). Odds ratios (ORs) and 95% confidence intervals (CIs) were then calculated.

**Results:**

All‐cause mortality [OR 1.54 (95% CI): (1.32–1.80)] and need for blood transfusion (OR 1.37; 95% CI 1.17–1.61) were higher among patients with moderate thrombocytopenia. However, adverse events, including post‐ERCP bleeding (OR 0.96; 95% CI 0.72–1.3), post‐ERCP pancreatitis (OR 0.81; 95% CI 0.69–1.02), sepsis (OR 1.04; 95% CI 0.93–1.16), and AKI (OR 1.11; 95% CI 1.00–1.23), were statistically not significant between the two groups.

**Conclusion:**

The cirrhotic population with moderate thrombocytopenia undergoing ERCP for choledocholithiasis experiences significantly worse outcomes compared to those with mild thrombocytopenia.

## 1 Introduction

Patients with liver cirrhosis face a heightened risk of complications during endoscopic procedures due to factors such as liver dysfunction, coagulopathy, portal hypertension, and other related challenges [1, 2]. Endoscopic retrograde cholangiopancreatography (ERCP), a frequently performed yet high‐risk intervention, carries an even greater likelihood of adverse events in this population [3]. Those with cirrhosis undergoing surgical procedures are significantly more prone to perioperative morbidity and mortality, with the extent of hepatic dysfunction acting as a critical determinant of outcomes [4]. Thrombocytopenia is a common and clinically significant complication of liver cirrhosis, arising from various factors such as splenic sequestration, decreased thrombopoietin production, and bone marrow suppression [5]. The severity of thrombocytopenia has been recognized as a valuable prognostic marker in cirrhotic patients, as severe thrombocytopenia (< 50 × 10^9^/L) can notably contribute to morbidity in liver disease [5, 6]. ERCP is routinely performed on patients with cirrhosis for the management of biliary pathologies such as choledocholithiasis [7]. However, thrombocytopenia complicates procedural risk assessment and post‐procedural management. The severity of thrombocytopenia—ranging from mild to moderate—can significantly affect post‐ERCP outcomes, including mortality and adverse events. While existing literature has discussed general procedural risks in cirrhotic populations, there is a lack of data specifically evaluating how varying degrees of thrombocytopenia influence outcomes in cirrhotic patients undergoing an ERCP.

This study seeks to bridge this knowledge gap by analyzing post‐ERCP outcomes in a large cohort of cirrhotic patients with mild versus moderate thrombocytopenia, using propensity score matching to adjust for confounding variables. By clarifying these associations, we aim to inform risk stratification and improve peri‐procedural planning for patients with liver cirrhosis undergoing ERCP.

## 2 Methods

### 2.1 Study Population and Design

This retrospective, propensity‐matched cohort study uses data from the TRINETX Research Network (Cambridge, MA, USA), which comprises 108 million patients from 62 healthcare organizations across the United States. We identified patients aged 18 years and older with cirrhosis who were admitted between January 2017 and February 2024. All data used in this study are de‐identified and anonymized, with no patient, institutional, or geographical identifiers. The 62 contributing healthcare organizations comprise academic medical centers and community hospitals. Data were collected from electronic health records (EHRs) and insurance claims, with a focus on inpatient settings to better reflect real‐world clinical scenarios and enhance generalizability.

A complete description of the information regarding the data source, study definition, inclusion criteria, and exclusion criteria used to query the TRINETX Research Network database, as well as their International Classification of Diseases, 10th Revision (ICD‐10), and Current Procedural Terminology (CPT) codes, is provided in the Supporting Information.

### 2.2 Ethics Statement and Consent

To protect patient confidentiality and autonomy, all data are de‐identified and anonymized, with no patient, institutional, or geographic identifiers retained. No informed patient consent or IRB review is needed for this study.

### 2.3 Study Cohort Selection

#### 2.3.1 Inclusion Criteria

Patients aged 18 years or older with cirrhosis undergoing ERCP for choledocholithiasis were included. Patients were divided into two groups: (I) ERCP + mild thrombocytopenia (platelet count, 101 000–150 000 cells/μL); (II) ERCP + moderate thrombocytopenia (platelet count, 30 000–100 000 cells/μL) (Supporting Information).

#### 2.3.2 Primary Outcomes

Our primary objective was to determine the difference in 30‐day all‐cause mortality between the two study groups.

#### 2.3.3 Secondary Outcomes

Secondary outcomes included post‐ERCP bleeding, post‐ERCP pancreatitis (PEP), abdominal pain, bile duct perforation, need for blood transfusion, sepsis, and other complications such as acute kidney injury (AKI).

### 2.4 Statistical Analysis

We employed the propensity score‐matching algorithm to create cohorts with matched demographics and baseline characteristics, thereby reducing selection bias and controlling for confounding factors. We used Trinetx's built‐in propensity score‐matching algorithm to match the two cohorts at a 1:1 ratio. Propensity score matching was conducted on 15 characteristics, including demographics (age, gender, race), presence of ascites, hepatic encephalopathy, esophageal varices, laboratory parameters (PT, aPTT, INR, hemoglobin, hematocrit, total bilirubin, creatinine, use of anticoagulants and antiplatelets, and serum sodium), and body mass index (BMI) (Supporting Information). The standardized difference evaluated the effectiveness of the propensity‐matching and assessed the balance of baseline demographic, laboratory, and clinical characteristics across the propensity‐matched cohorts. Generally, a standardized difference of less than 0.1 is considered minimal, indicating adequate propensity score matching.

**FIGURE 1:**
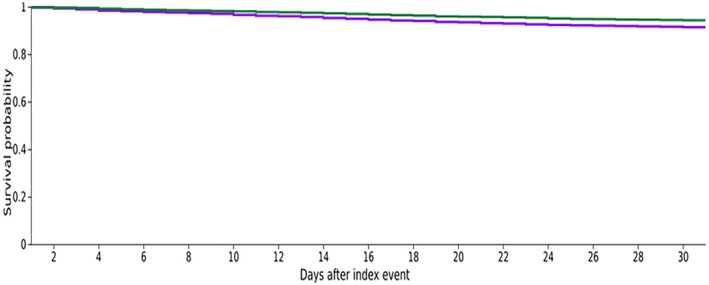
Kaplan–Meier survival curves demonstrating 30‐day survival probability in both study groups [HR (95% CI): 1.52 (1.304–1.78), log‐rank test *p* value < 0.0001].

We compared baseline demographic, laboratory, and clinical characteristics across the propensity‐matched cohorts using either the Chi‐square/Fisher's exact test or the independent‐samples t‐test. After propensity score matching, we calculated odds ratios (ORs) along with 95% confidence intervals (CIs) to assess outcomes between the two groups. Additionally, we employed Kaplan–Meier curves to illustrate 1‐month survival outcomes in both study groups and calculated ORs along with 95% CIs, as shown in Figure 1.

## 3 Results

We conducted a comprehensive comparative analysis of ERCP outcomes for bile duct choledocholithiasis in patients with liver cirrhosis. The patients were divided into two groups based on platelet count at the time of ERCP. After propensity score matching, 5097 patients in each group were analyzed for the primary and secondary outcomes of ERCP, enabling more accurate comparisons of demographic variables.

Table 1 presents patient counts and demographic characteristics before and after propensity score matching, indicating that the procedure achieved balance in age, sex, ethnicity, and gender across the matched groups. Table 2 outlines the possible etiologies of cirrhosis.

**TABLE 1:** Essential characteristics of the patients in both groups before and after propensity matching.

Study cohorts	Demographics characteristics					
		Mean ± SD	Patients	% of Cohort	*p*	Std diff.
1 2	Age at Index	59.0 ± 13.6 59.4 ± 13.5	5097 5097	100% 100%	0.133	0.030
1 2	White		3812 3809	74.8% 74.7%	0.945	0.001
1 2	American Indian or Alaska Native		38 33	0.7% 0.6%	0.552	0.012
1 2	Female		1898 1877	37.2% 36.8%	0.667	0.009
1 2	Native Hawaiian or Other Pacific Islander		18 23	0.4% 0.5%	0.434	0.015
1 2	Black or African American		533 563	10.5% 11.0%	0.337	0.019
1 2	Male		3199 3220	62.8% 63.2%	0.667	0.009
1 2	Other Race		225 221	4.4% 4.3%	0.846	0.004
1 2	Asian		216 222	4.2% 4.4%	0.769	0.006

*Note:* Cohort A: moderate thrombocytopenia; Cohort B: mild thrombocytopenia.

**TABLE 2:** Different etiologies of the cirrhosis.

Cohort	Diagnosis					
		Mean ± SD	Patients	% of Cohort	*p*	Std diff.
1 2	Biliary cirrhosis, unspecified		470 437	9.2% 8.6%	0.251	0.023
1 2	Alcoholic cirrhosis of liver		1388 1447	27.2% 28.4%	0.192	0.026
1 2	Other and unspecified cirrhosis of liver		3505 3618	68.8% 71.0%	0.015	0.048
1 2	Other cirrhosis of liver		2334 2344	45.8% 46.0%	0.842	0.004
1 2	Alcoholic cirrhosis of liver without ascites		1182 1226	23.2% 24.1%	0.305	0.020
1 2	Alcoholic cirrhosis of liver with ascites		832 854	16.3% 16.8%	0.558	0.012
1 2	Toxic liver disease with fibrosis and cirrhosis of liver		26 22	0.5% 0.4%	0.563	0.011
1 2	Chronic viral hepatitis		823 829	16.1% 16.3%	0.872	0.003
1 2	Nonalcoholic steatohepatitis (NASH)		718 747	14.1% 14.7%	0.413	0.016

### 3.1 All‐Cause Mortality

Compared to the group with mild thrombocytopenia, all‐cause mortality was significantly higher among patients with moderate thrombocytopenia [OR (95% CI): 1.54 (1.32–1.80)], Table 3. Kaplan–Meier survival curves demonstrate the 30‐day survival probability in both study groups [HR (95% CI): 1.52 (1.30–1.78); log‐rank test *p* value < 0.0001; Figure 1].

**TABLE 3:** Primary and secondary outcomes in cirrhotic patients with thrombocytopenia post‐ERCP for choledocholithiasis.

Outcomes	ERCP + Cirrhotic patients with moderate thrombocytopenia	ERCP + Cirrhotic patients with mild thrombocytopenia	Odds ratio	95% Cl
	*n* = 5097	*n* = 5097		
Primary outcomes				
All‐cause mortality	440 (8.6%)	294 (5.7%)	1.54	1.32–1.80
Secondary outcomes				
Post‐ERCP bleeding	90 (1.7%)	93 (1.8%)	0.96	0.72–1.29
Post‐ERCP pancreatitis	137 (2.6%)	163 (3.1%)	0.81	0.64–1.026
Need for blood product transfusion	369 (7.2%)	274 (5.3%)	1.37	1.17–1.61
Perforation of the bile duct	0 (0.0%)	0 (0.0%)	—	—
Sepsis	769 (15.08%)	739 (14.5%)	1.04	0.93–1.16
Acute kidney injury	991 (19.44%)	907 (17.8%)	1.115	1.0–1.23
Jaundice	487 (9.5%)	526 (10.31%)	0.91	0.80–1.045

### 3.2 Secondary Outcomes

Patients with moderate thrombocytopenia have a higher need for blood transfusion (OR 1.37; 95% CI 1.17–1.61) during ERCP than those with mild thrombocytopenia. However, adverse events, including post‐ERCP bleeding (OR 0.96; 95% CI 0.72–1.3), PEP (OR 0.81; 95% CI 0.69–1.02), sepsis (OR 1.04; 95% CI 0.93–1.16), and AKI (OR 1.11; 95% CI 1.00–1.23), were statistically not significant between the two groups. Furthermore, there was no difference in the other outcomes, such as jaundice, abdominal pain, and bile duct perforation, between the two groups.

Table 3 compares the primary and secondary outcomes of ERCP between the two groups.

## 4 Discussion

Our study underscores the influence of thrombocytopenia severity on post‐ERCP outcomes in cirrhotic patients. It indicates that cirrhotic patients with moderate thrombocytopenia face a higher risk of mortality and the necessity for blood transfusion, compared to matched controls with mild thrombocytopenia.

Thrombocytopenia is a common laboratory finding in patients with liver cirrhosis [8]. Its multifactorial pathogenesis includes splenic sequestration of platelets secondary to portal hypertension, myelosuppression, decreased thrombopoietin (TPO) production, and increased peripheral destruction [9]. Although clinically significant spontaneous bleeding due to thrombocytopenia is rare, its presence carries important clinical implications for cirrhotic patients, such as an increased risk of infections, procedure‐related bleeding, and mortality, as observed in our study. Thrombocytopenia is often used as a marker to predict the severity of cirrhosis and frequently complicates the performance of invasive procedures among cirrhotic patients [10, 11, 12].

ERCP is one of the most performed endoscopic procedures and carries certain risks, including PEP, post‐ERCP bleeding, infections, perforation, and anesthesia‐related events [1]. These risks are heightened among cirrhotic patients due to compromised synthetic liver function, portal hypertension, underlying coagulopathy, varices, and hepatic encephalopathy [13]. A systematic review and meta‐analysis by Harmeet Singh et al. on ERCP‐related adverse events indicated a higher risk of post‐ERCP hemorrhage (pooled OR 2.05 [95% CI: 1.62–2.58, *p* < 0.01, *I* ^2^ = 2.1%]) and PEP (pooled OR 1.33 [95% CI: 1.04–1.70, *p* = 0.021, *I* ^2^ = 65%]) in cirrhotic patients compared to non‐cirrhotic individuals [14]. A retrospective matched case–control study by Navaneethan et al. [13] using the 2010 National Inpatient Sample database also found a higher risk of ERCP‐associated hemorrhage, 2.1% in cirrhosis versus 1.2% in non‐cirrhosis patients. Strong evidence from the literature suggests that cirrhotic patients face a greater risk of developing post‐ERCP complications compared to the general population, with this risk further increasing among individuals with more severe disease and in cases of decompensated cirrhosis [15, 16, 17]. Likewise, several studies have reported higher rates of post‐ERCP bleeding in Child‐Pugh (CP) class C compared to classes A and B [18, 19, 20].

Despite existing literature, there is a lack of studies assessing the role of underlying coagulopathy or thrombocytopenia as potential predictors of adverse post‐ERCP outcomes in cirrhotic patients. Our study aims to evaluate the association between thrombocytopenia and post‐ERCP‐related outcomes. Authors observed higher odds of mortality and the need for blood products among cirrhotic patients with moderate thrombocytopenia compared to controls (those with mild thrombocytopenia), indicating the potential importance of pre‐procedural risk stratification and correcting thrombocytopenia to a safer threshold before the procedure to prevent complications and lower in‐hospital mortality. The severity of thrombocytopenia could act as an independent predictor of adverse clinical outcomes in this population, and clinicians should consider the clinical implications of thrombocytopenia while planning invasive procedures for cirrhotic patients.

Our study has certain limitations as well. It is restricted to patients who underwent ERCP to remove bile duct calculi, which can lead to complications such as cholangitis. Additionally, this study did not evaluate whether the effect of underlying thrombocytopenia varies when comparing cirrhotic patients with a matched, relatively healthy cohort. Moreover, our study did not differentiate ERCP outcomes among patients with compensated versus non‐compensated cirrhosis. Additionally, due to limitations in the available dataset, we were unable to obtain MELD‐Na scores for all patients included in the study. Finally, this study did not explore the influence of other underlying coagulopathies as potential predictors of adverse post‐procedure outcomes. Further research is needed to identify potential predictors that may heighten the risk of unfavorable clinical and post‐procedural outcomes in this vulnerable population.

## 5 Conclusions

This study indicates an increased mortality risk among cirrhotic patients with moderate thrombocytopenia who underwent ERCP. Furthermore, patients with moderate thrombocytopenia were more likely to require blood transfusions.

## Funding

The authors have nothing to report.

## Ethics Statement

All methods were conducted in accordance with the ethical standards of the institutional and/or national research committee and with the 1964 Helsinki Declaration and its later amendments.

## Consent

This study used de‐identified patient data from the TriNetX research network and was exempt from institutional review board (IRB) oversight.

## Conflicts of Interest

The authors declare no conflicts of interest.

## Supporting information


**Data S1:** jgh370418‐sup‐0001‐Supinfo.docx.

## Data Availability

The data that support the findings of this study are available from TriNetX. Restrictions apply to the availability of these data, which were used under license for this study. Data are available from the authors upon reasonable request and with permission of TriNetX (https://trinetx.com).
