# Supplementary material for: Post‐ERCP Outcomes in Cirrhotic Patients With Thrombocytopenia: A Propensity‐Matched Retrospective Comparative Analysis on TriNetX Health Research Database
Source: JGH Open. 2026 May 20;10(5):e70418. doi: 10.1002/jgh3.70418 (PMC13240326; doi:10.1002/jgh3.70418)
Supplement: Supplementary file 1 — Data S1: jgh370418‐sup‐0001‐Supinfo.docx. [file JGH3-10-e70418-s001.docx]

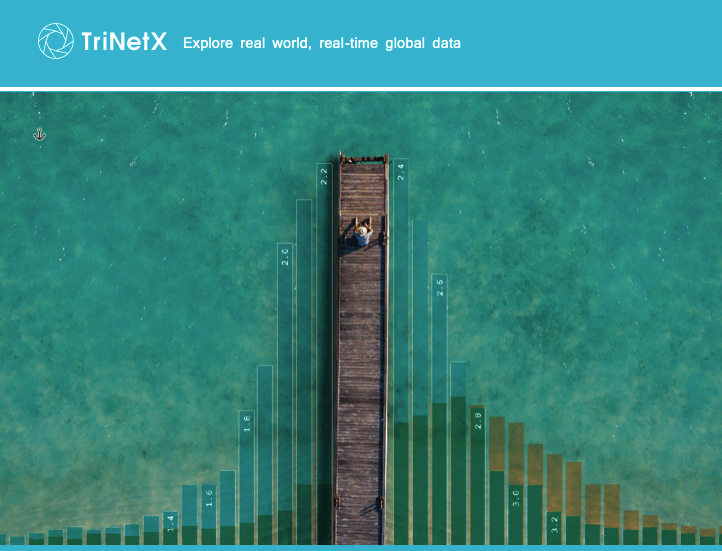


Hepatic Hydrothorax , Tips vs Thoracocentesis

Compare Outcomes Analysis

Created by TriNetX on Oct 11, 2025, 20:26:05 UTC

# Introduction

TriNetX is the global federated health research network providing access to electronic medical records (diagnoses, procedures, medications, laboratory values, genomic information) across large healthcare organizations (HCOs). This report was run on the set of HCOs grouped into a network called Global Collaborative Network. This network included 155 HCO(s).

This report describes a Compare Outcomes Analysis, named Unnamed Analysis, generated by the TriNetX platform on Oct 11, 2025, 20:26:05 UTC. This analysis compared the outcomes of two cohorts: Cohort A (4,534 patients) named ERCP-Group A and Cohort B (12,666 patients) named ERCP-Group B.

This analysis was run by Azhar Hussain (hussaiaz@upstate.edu) and downloaded by Azhar Hussain (hussaiaz@upstate.edu).

# Methods

The analysis process includes two main steps: 1) Defining the cohorts through query criteria; 2) Setting up and running the analysis. Setting up the analysis requires definitions for the index event, outcomes criteria, and the time frame. Compare outcomes supports four analyses: Measures of Association, Survival, Number of Instances and Lab result distribution. These analyses have additional options that are listed in the Outcomes Definitions and Analyses Specifications section below. Furthermore, characteristics of the cohorts that are balanced using propensity score matching are also included in the Propensity Score Matching section.

## Cohorts definition

This section lists all terms used in the definitions of the two cohorts.

### Query Criteria for Cohort 1 (query name: ERCP-Group A)

This query was run on the network Global Collaborative Network with 155 HCO(s) queried and 154 HCO(s) responded. A total of 52 provider(s) responded with patients. The final cohort included 4,534 patients who matched the query criteria listed in the table below. For the text representation of the query criteria please see Appendix A.

|  | | | | | |
| --- | --- | --- | --- | --- | --- |
| Ungrouped terms | | | | | |
|  | must have |  | demographics | Age | Age (between 18 and 89 years (most recent occurrence)) |
|  |  | and any of | diagnosis | UMLS:ICD10CM:K74.60 | Unspecified cirrhosis of liver |
|  |  |  | diagnosis | UMLS:ICD10CM:K74.5 | Biliary cirrhosis, unspecified |
|  |  |  | diagnosis | UMLS:ICD10CM:K71.7 | Toxic liver disease with fibrosis and cirrhosis of liver |
|  |  |  | diagnosis | UMLS:ICD10CM:K74.3 | Primary biliary cirrhosis |
|  |  |  | diagnosis | UMLS:ICD10CM:K74.4 | Secondary biliary cirrhosis |
|  |  |  | diagnosis | UMLS:ICD10CM:K74.6 | Other and unspecified cirrhosis of liver |
|  |  |  | diagnosis | UMLS:ICD10CM:K70.11 | Alcoholic hepatitis with ascites |
|  |  |  | diagnosis | UMLS:ICD10CM:K76.1 | Chronic passive congestion of liver |
|  |  |  | diagnosis | UMLS:ICD10CM:K70.30 | Alcoholic cirrhosis of liver without ascites |
|  |  |  | diagnosis | UMLS:ICD10CM:K70.30 | Alcoholic cirrhosis of liver without ascites |
|  |  |  | diagnosis | UMLS:ICD10CM:K74.3 | Primary biliary cirrhosis |
|  |  |  | diagnosis | UMLS:ICD10CM:K70.31 | Alcoholic cirrhosis of liver with ascites |
|  |  |  | diagnosis | UMLS:ICD10CM:K74.00 | Hepatic fibrosis, unspecified |
| Group 1 | | | | | |
|  | **Group 1A** | | | | |
|  | must have | any of | diagnosis | UMLS:ICD10CM:K80.50 | Calculus of bile duct without cholangitis or cholecystitis without obstruction |
|  |  |  | diagnosis | UMLS:ICD10CM:K80.5 | Calculus of bile duct without cholangitis or cholecystitis |
|  |  |  | diagnosis | UMLS:ICD10CM:K83.0 | Cholangitis |
|  |  |  | diagnosis | UMLS:ICD10CM:K80.4 | Calculus of bile duct with cholecystitis |
|  |  |  | diagnosis | UMLS:ICD10CM:K80.51 | Calculus of bile duct without cholangitis or cholecystitis with obstruction |
|  |  |  | diagnosis | UMLS:ICD10CM:K80.42 | Calculus of bile duct with acute cholecystitis without obstruction |
|  |  |  | diagnosis | UMLS:ICD10CM:K80.44 | Calculus of bile duct with chronic cholecystitis without obstruction |
|  |  |  | diagnosis | UMLS:ICD10CM:K80.40 | Calculus of bile duct with cholecystitis, unspecified, without obstruction |
|  |  |  | diagnosis | UMLS:ICD10CM:K80.43 | Calculus of bile duct with acute cholecystitis with obstruction |
|  |  |  | diagnosis | UMLS:ICD10CM:K80.45 | Calculus of bile duct with chronic cholecystitis with obstruction |
|  |  |  | diagnosis | UMLS:ICD10CM:K80.41 | Calculus of bile duct with cholecystitis, unspecified, with obstruction |
|  |  |  | diagnosis | UMLS:ICD10CM:K80.70 | Calculus of gallbladder and bile duct without cholecystitis without obstruction |
|  |  |  | diagnosis | UMLS:ICD10CM:K80.71 | Calculus of gallbladder and bile duct without cholecystitis with obstruction |
|  |  |  | diagnosis | UMLS:ICD10CM:K80.62 | Calculus of gallbladder and bile duct with acute cholecystitis without obstruction |
|  |  |  | diagnosis | UMLS:ICD10CM:K80.64 | Calculus of gallbladder and bile duct with chronic cholecystitis without obstruction |
|  |  |  | diagnosis | UMLS:ICD10CM:K80.63 | Calculus of gallbladder and bile duct with acute cholecystitis with obstruction |
|  |  |  | diagnosis | UMLS:ICD10CM:K80.66 | Calculus of gallbladder and bile duct with acute and chronic cholecystitis without obstruction |
|  |  |  | diagnosis | UMLS:ICD10CM:K80.67 | Calculus of gallbladder and bile duct with acute and chronic cholecystitis with obstruction |
|  |  |  | diagnosis | UMLS:ICD10CM:K80.65 | Calculus of gallbladder and bile duct with chronic cholecystitis with obstruction |
|  | date constraint | | The terms in this group occurred at any time | | |
|  | event relationship | | Any instance of Group 1B occurred within 1 day before or up to 7 days after any instance of Group 1A | | |
|  | **Group 1B** | | | | |
|  | must have | any of | procedure | UMLS:CPT:43264 | Endoscopic retrograde cholangiopancreatography (ERCP); with removal of calculi/debris from biliary/pancreatic duct(s) |
|  |  |  | procedure | UMLS:CPT:43274 | Endoscopic retrograde cholangiopancreatography (ERCP); with placement of endoscopic stent into biliary or pancreatic duct, including pre- and post-dilation and guide wire passage, when performed, including sphincterotomy, when performed, each stent |
|  |  |  | procedure | UMLS:CPT:43273 | Endoscopic cannulation of papilla with direct visualization of pancreatic/common bile duct(s) (List separately in addition to code(s) for primary procedure) |
|  |  |  | procedure | UMLS:CPT:43260 | Endoscopic retrograde cholangiopancreatography (ERCP); diagnostic, including collection of specimen(s) by brushing or washing, when performed (separate procedure) |
|  |  |  | procedure | UMLS:CPT:43261 | Endoscopic retrograde cholangiopancreatography (ERCP); with biopsy, single or multiple |
|  |  |  | procedure | UMLS:CPT:43262 | Endoscopic retrograde cholangiopancreatography (ERCP); with sphincterotomy/papillotomy |
|  |  |  | procedure | UMLS:CPT:43263 | Endoscopic retrograde cholangiopancreatography (ERCP); with pressure measurement of sphincter of Oddi |
|  |  |  | procedure | UMLS:CPT:43264 | Endoscopic retrograde cholangiopancreatography (ERCP); with removal of calculi/debris from biliary/pancreatic duct(s) |
|  |  |  | procedure | UMLS:CPT:43265 | Endoscopic retrograde cholangiopancreatography (ERCP); with destruction of calculi, any method (eg, mechanical, electrohydraulic, lithotripsy) |
|  |  |  | procedure | UMLS:CPT:43273 | Endoscopic cannulation of papilla with direct visualization of pancreatic/common bile duct(s) (List separately in addition to code(s) for primary procedure) |
|  |  |  | procedure | UMLS:CPT:43274 | Endoscopic retrograde cholangiopancreatography (ERCP); with placement of endoscopic stent into biliary or pancreatic duct, including pre- and post-dilation and guide wire passage, when performed, including sphincterotomy, when performed, each stent |
|  |  |  | procedure | UMLS:CPT:43275 | Endoscopic retrograde cholangiopancreatography (ERCP); with removal of foreign body(s) or stent(s) from biliary/pancreatic duct(s) |
|  |  |  | procedure | UMLS:CPT:43276 | Endoscopic retrograde cholangiopancreatography (ERCP); with removal and exchange of stent(s), biliary or pancreatic duct, including pre- and post-dilation and guide wire passage, when performed, including sphincterotomy, when performed, each stent exchanged |
|  |  |  | procedure | UMLS:CPT:43277 | Endoscopic retrograde cholangiopancreatography (ERCP); with trans-endoscopic balloon dilation of biliary/pancreatic duct(s) or of ampulla (sphincteroplasty), including sphincterotomy, when performed, each duct |
|  |  |  | procedure | UMLS:CPT:43278 | Endoscopic retrograde cholangiopancreatography (ERCP); with ablation of tumor(s), polyp(s), or other lesion(s), including pre- and post-dilation and guide wire passage, when performed |
|  |  | and | laboratory | TNX:9020 | Platelets [#/volume] in Blood (between 30.00 and 100.00 10*3/uL; at least 18 years old at event) |

### Query Criteria for Cohort 2 (query name: ERCP-Group B)

This query was run on the network Global Collaborative Network with 156 HCO(s) queried and 156 HCO(s) responded. A total of 55 provider(s) responded with patients. The final cohort included 12,666 patients who matched the query criteria listed in the table below.

| Ungrouped terms | | | | | |
| --- | --- | --- | --- | --- | --- |
|  | must have |  | demographics | Age | Age (between 18 and 89 years (most recent occurrence)) |
|  |  | and any of | diagnosis | UMLS:ICD10CM:K74.60 | Unspecified cirrhosis of liver |
|  |  |  | diagnosis | UMLS:ICD10CM:K74.5 | Biliary cirrhosis, unspecified |
|  |  |  | diagnosis | UMLS:ICD10CM:K71.7 | Toxic liver disease with fibrosis and cirrhosis of liver |
|  |  |  | diagnosis | UMLS:ICD10CM:K74.3 | Primary biliary cirrhosis |
|  |  |  | diagnosis | UMLS:ICD10CM:K74.4 | Secondary biliary cirrhosis |
|  |  |  | diagnosis | UMLS:ICD10CM:K74.6 | Other and unspecified cirrhosis of liver |
|  |  |  | diagnosis | UMLS:ICD10CM:K70.11 | Alcoholic hepatitis with ascites |
|  |  |  | diagnosis | UMLS:ICD10CM:K76.1 | Chronic passive congestion of liver |
|  |  |  | diagnosis | UMLS:ICD10CM:K70.30 | Alcoholic cirrhosis of liver without ascites |
|  |  |  | diagnosis | UMLS:ICD10CM:K70.30 | Alcoholic cirrhosis of liver without ascites |
|  |  |  | diagnosis | UMLS:ICD10CM:K74.3 | Primary biliary cirrhosis |
|  |  |  | diagnosis | UMLS:ICD10CM:K70.31 | Alcoholic cirrhosis of liver with ascites |
|  |  |  | diagnosis | UMLS:ICD10CM:K74.00 | Hepatic fibrosis, unspecified |
| Group 1 | | | | | |
|  | **Group 1A** | | | | |
|  | must have | any of | diagnosis | UMLS:ICD10CM:K80.50 | Calculus of bile duct without cholangitis or cholecystitis without obstruction |
|  |  |  | diagnosis | UMLS:ICD10CM:K80.5 | Calculus of bile duct without cholangitis or cholecystitis |
|  |  |  | diagnosis | UMLS:ICD10CM:K83.0 | Cholangitis |
|  |  |  | diagnosis | UMLS:ICD10CM:K80.4 | Calculus of bile duct with cholecystitis |
|  |  |  | diagnosis | UMLS:ICD10CM:K80.51 | Calculus of bile duct without cholangitis or cholecystitis with obstruction |
|  |  |  | diagnosis | UMLS:ICD10CM:K80.42 | Calculus of bile duct with acute cholecystitis without obstruction |
|  |  |  | diagnosis | UMLS:ICD10CM:K80.44 | Calculus of bile duct with chronic cholecystitis without obstruction |
|  |  |  | diagnosis | UMLS:ICD10CM:K80.40 | Calculus of bile duct with cholecystitis, unspecified, without obstruction |
|  |  |  | diagnosis | UMLS:ICD10CM:K80.43 | Calculus of bile duct with acute cholecystitis with obstruction |
|  |  |  | diagnosis | UMLS:ICD10CM:K80.45 | Calculus of bile duct with chronic cholecystitis with obstruction |
|  |  |  | diagnosis | UMLS:ICD10CM:K80.41 | Calculus of bile duct with cholecystitis, unspecified, with obstruction |
|  |  |  | diagnosis | UMLS:ICD10CM:K80.70 | Calculus of gallbladder and bile duct without cholecystitis without obstruction |
|  |  |  | diagnosis | UMLS:ICD10CM:K80.71 | Calculus of gallbladder and bile duct without cholecystitis with obstruction |
|  |  |  | diagnosis | UMLS:ICD10CM:K80.62 | Calculus of gallbladder and bile duct with acute cholecystitis without obstruction |
|  |  |  | diagnosis | UMLS:ICD10CM:K80.64 | Calculus of gallbladder and bile duct with chronic cholecystitis without obstruction |
|  |  |  | diagnosis | UMLS:ICD10CM:K80.63 | Calculus of gallbladder and bile duct with acute cholecystitis with obstruction |
|  |  |  | diagnosis | UMLS:ICD10CM:K80.66 | Calculus of gallbladder and bile duct with acute and chronic cholecystitis without obstruction |
|  |  |  | diagnosis | UMLS:ICD10CM:K80.67 | Calculus of gallbladder and bile duct with acute and chronic cholecystitis with obstruction |
|  |  |  | diagnosis | UMLS:ICD10CM:K80.65 | Calculus of gallbladder and bile duct with chronic cholecystitis with obstruction |
|  | date constraint | | The terms in this group occurred at any time | | |
|  | event relationship | | Any instance of Group 1B occurred within 1 day before or up to 7 days after any instance of Group 1A | | |
|  | **Group 1B** | | | | |
|  | must have | any of | procedure | UMLS:CPT:43264 | Endoscopic retrograde cholangiopancreatography (ERCP); with removal of calculi/debris from biliary/pancreatic duct(s) |
|  |  |  | procedure | UMLS:CPT:43274 | Endoscopic retrograde cholangiopancreatography (ERCP); with placement of endoscopic stent into biliary or pancreatic duct, including pre- and post-dilation and guide wire passage, when performed, including sphincterotomy, when performed, each stent |
|  |  |  | procedure | UMLS:CPT:43273 | Endoscopic cannulation of papilla with direct visualization of pancreatic/common bile duct(s) (List separately in addition to code(s) for primary procedure) |
|  |  |  | procedure | UMLS:CPT:43260 | Endoscopic retrograde cholangiopancreatography (ERCP); diagnostic, including collection of specimen(s) by brushing or washing, when performed (separate procedure) |
|  |  |  | procedure | UMLS:CPT:43261 | Endoscopic retrograde cholangiopancreatography (ERCP); with biopsy, single or multiple |
|  |  |  | procedure | UMLS:CPT:43262 | Endoscopic retrograde cholangiopancreatography (ERCP); with sphincterotomy/papillotomy |
|  |  |  | procedure | UMLS:CPT:43263 | Endoscopic retrograde cholangiopancreatography (ERCP); with pressure measurement of sphincter of Oddi |
|  |  |  | procedure | UMLS:CPT:43264 | Endoscopic retrograde cholangiopancreatography (ERCP); with removal of calculi/debris from biliary/pancreatic duct(s) |
|  |  |  | procedure | UMLS:CPT:43265 | Endoscopic retrograde cholangiopancreatography (ERCP); with destruction of calculi, any method (eg, mechanical, electrohydraulic, lithotripsy) |
|  |  |  | procedure | UMLS:CPT:43273 | Endoscopic cannulation of papilla with direct visualization of pancreatic/common bile duct(s) (List separately in addition to code(s) for primary procedure) |
|  |  |  | procedure | UMLS:CPT:43274 | Endoscopic retrograde cholangiopancreatography (ERCP); with placement of endoscopic stent into biliary or pancreatic duct, including pre- and post-dilation and guide wire passage, when performed, including sphincterotomy, when performed, each stent |
|  |  |  | procedure | UMLS:CPT:43275 | Endoscopic retrograde cholangiopancreatography (ERCP); with removal of foreign body(s) or stent(s) from biliary/pancreatic duct(s) |
|  |  |  | procedure | UMLS:CPT:43276 | Endoscopic retrograde cholangiopancreatography (ERCP); with removal and exchange of stent(s), biliary or pancreatic duct, including pre- and post-dilation and guide wire passage, when performed, including sphincterotomy, when performed, each stent exchanged |
|  |  |  | procedure | UMLS:CPT:43277 | Endoscopic retrograde cholangiopancreatography (ERCP); with trans-endoscopic balloon dilation of biliary/pancreatic duct(s) or of ampulla (sphincteroplasty), including sphincterotomy, when performed, each duct |
|  |  |  | procedure | UMLS:CPT:43278 | Endoscopic retrograde cholangiopancreatography (ERCP); with ablation of tumor(s), polyp(s), or other lesion(s), including pre- and post-dilation and guide wire passage, when performed |
|  |  | and | laboratory | TNX:9020 | Platelets [#/volume] in Blood (at least 100.00 10*3/uL; at least 18 years old at event) |

## Analysis Setup

This section contains the Index Event and Time Window definitions and a list of selected outcomes and the analyses.

### Index Event & Time Window Definitions

The index event defines the point in time when each patient in the cohort enters the analysis. To define an index event for the cohort, one or more criteria for the cohort must be selected. The index date for each patient within a cohort is the day on which the patient first met the selected criteria for the cohort (listed in the table below).

As the index event defines the earliest time point after which outcomes are analyzed, the time window defines the duration during which outcomes are analyzed. The time window can start on the same day as the index event or at any specified time interval after the index event. The time window can end any time after the start date. Outcomes are defined as diagnoses, medications, procedures, or laboratory values that happened in the time window starting after the first occurrence of the index event.

### Time Window Used in this Analysis

This analysis included outcomes that occurred in the time window that started 1 day after the first occurrence of the index event and ended 30 days after the first occurrence of the index event.

The index event only includes events that occurred up to 20 years ago. Patients whose index event occurred 20 years or more ago are excluded. In this analysis, 0 patients in Cohort 1 and 0 patients in Cohort 2 were excluded because they met the index event more than 20 years ago.

### Index Events Used in this Analysis

Index events for the Compare Outcomes analysis were derived from the cohort definitions. Index events were defined separately for each cohort and were based on the criteria used in the original cohort definition. Please see Appendix B for the text representation of the index event definition.

The index event for Cohort 1 (query name: ERCP-Group A) was defined as the following:

|  | | | | | |
| --- | --- | --- | --- | --- | --- |
| Ungrouped terms | | | | | |
|  | must have | any of | diagnosis | UMLS:ICD10CM:K74.60 | Unspecified cirrhosis of liver |
|  |  |  | diagnosis | UMLS:ICD10CM:K74.5 | Biliary cirrhosis, unspecified |
|  |  |  | diagnosis | UMLS:ICD10CM:K71.7 | Toxic liver disease with fibrosis and cirrhosis of liver |
|  |  |  | diagnosis | UMLS:ICD10CM:K74.3 | Primary biliary cirrhosis |
|  |  |  | diagnosis | UMLS:ICD10CM:K74.4 | Secondary biliary cirrhosis |
|  |  |  | diagnosis | UMLS:ICD10CM:K74.6 | Other and unspecified cirrhosis of liver |
|  |  |  | diagnosis | UMLS:ICD10CM:K70.11 | Alcoholic hepatitis with ascites |
|  |  |  | diagnosis | UMLS:ICD10CM:K76.1 | Chronic passive congestion of liver |
|  |  |  | diagnosis | UMLS:ICD10CM:K70.30 | Alcoholic cirrhosis of liver without ascites |
|  |  |  | diagnosis | UMLS:ICD10CM:K70.30 | Alcoholic cirrhosis of liver without ascites |
|  |  |  | diagnosis | UMLS:ICD10CM:K74.3 | Primary biliary cirrhosis |
|  |  |  | diagnosis | UMLS:ICD10CM:K70.31 | Alcoholic cirrhosis of liver with ascites |
|  |  |  | diagnosis | UMLS:ICD10CM:K74.00 | Hepatic fibrosis, unspecified |
| Group 1 | | | | | |
|  | **Group 1A** | | | | |
|  | must have | any of | diagnosis | UMLS:ICD10CM:K80.50 | Calculus of bile duct without cholangitis or cholecystitis without obstruction |
|  |  |  | diagnosis | UMLS:ICD10CM:K80.5 | Calculus of bile duct without cholangitis or cholecystitis |
|  |  |  | diagnosis | UMLS:ICD10CM:K83.0 | Cholangitis |
|  |  |  | diagnosis | UMLS:ICD10CM:K80.4 | Calculus of bile duct with cholecystitis |
|  |  |  | diagnosis | UMLS:ICD10CM:K80.51 | Calculus of bile duct without cholangitis or cholecystitis with obstruction |
|  |  |  | diagnosis | UMLS:ICD10CM:K80.42 | Calculus of bile duct with acute cholecystitis without obstruction |
|  |  |  | diagnosis | UMLS:ICD10CM:K80.44 | Calculus of bile duct with chronic cholecystitis without obstruction |
|  |  |  | diagnosis | UMLS:ICD10CM:K80.40 | Calculus of bile duct with cholecystitis, unspecified, without obstruction |
|  |  |  | diagnosis | UMLS:ICD10CM:K80.43 | Calculus of bile duct with acute cholecystitis with obstruction |
|  |  |  | diagnosis | UMLS:ICD10CM:K80.45 | Calculus of bile duct with chronic cholecystitis with obstruction |
|  |  |  | diagnosis | UMLS:ICD10CM:K80.41 | Calculus of bile duct with cholecystitis, unspecified, with obstruction |
|  |  |  | diagnosis | UMLS:ICD10CM:K80.70 | Calculus of gallbladder and bile duct without cholecystitis without obstruction |
|  |  |  | diagnosis | UMLS:ICD10CM:K80.71 | Calculus of gallbladder and bile duct without cholecystitis with obstruction |
|  |  |  | diagnosis | UMLS:ICD10CM:K80.62 | Calculus of gallbladder and bile duct with acute cholecystitis without obstruction |
|  |  |  | diagnosis | UMLS:ICD10CM:K80.64 | Calculus of gallbladder and bile duct with chronic cholecystitis without obstruction |
|  |  |  | diagnosis | UMLS:ICD10CM:K80.63 | Calculus of gallbladder and bile duct with acute cholecystitis with obstruction |
|  |  |  | diagnosis | UMLS:ICD10CM:K80.66 | Calculus of gallbladder and bile duct with acute and chronic cholecystitis without obstruction |
|  |  |  | diagnosis | UMLS:ICD10CM:K80.67 | Calculus of gallbladder and bile duct with acute and chronic cholecystitis with obstruction |
|  |  |  | diagnosis | UMLS:ICD10CM:K80.65 | Calculus of gallbladder and bile duct with chronic cholecystitis with obstruction |
|  | date constraint | | The terms in this group occurred at any time | | |
|  | event relationship | | Any instance of Group 1B occurred within 1 day before or up to 7 days after any instance of Group 1A | | |
|  | **Group 1B** | | | | |
|  | must have | any of | procedure | UMLS:CPT:43264 | Endoscopic retrograde cholangiopancreatography (ERCP); with removal of calculi/debris from biliary/pancreatic duct(s) |
|  |  |  | procedure | UMLS:CPT:43274 | Endoscopic retrograde cholangiopancreatography (ERCP); with placement of endoscopic stent into biliary or pancreatic duct, including pre- and post-dilation and guide wire passage, when performed, including sphincterotomy, when performed, each stent |
|  |  |  | procedure | UMLS:CPT:43273 | Endoscopic cannulation of papilla with direct visualization of pancreatic/common bile duct(s) (List separately in addition to code(s) for primary procedure) |
|  |  |  | procedure | UMLS:CPT:43260 | Endoscopic retrograde cholangiopancreatography (ERCP); diagnostic, including collection of specimen(s) by brushing or washing, when performed (separate procedure) |
|  |  |  | procedure | UMLS:CPT:43261 | Endoscopic retrograde cholangiopancreatography (ERCP); with biopsy, single or multiple |
|  |  |  | procedure | UMLS:CPT:43262 | Endoscopic retrograde cholangiopancreatography (ERCP); with sphincterotomy/papillotomy |
|  |  |  | procedure | UMLS:CPT:43263 | Endoscopic retrograde cholangiopancreatography (ERCP); with pressure measurement of sphincter of Oddi |
|  |  |  | procedure | UMLS:CPT:43264 | Endoscopic retrograde cholangiopancreatography (ERCP); with removal of calculi/debris from biliary/pancreatic duct(s) |
|  |  |  | procedure | UMLS:CPT:43265 | Endoscopic retrograde cholangiopancreatography (ERCP); with destruction of calculi, any method (eg, mechanical, electrohydraulic, lithotripsy) |
|  |  |  | procedure | UMLS:CPT:43273 | Endoscopic cannulation of papilla with direct visualization of pancreatic/common bile duct(s) (List separately in addition to code(s) for primary procedure) |
|  |  |  | procedure | UMLS:CPT:43274 | Endoscopic retrograde cholangiopancreatography (ERCP); with placement of endoscopic stent into biliary or pancreatic duct, including pre- and post-dilation and guide wire passage, when performed, including sphincterotomy, when performed, each stent |
|  |  |  | procedure | UMLS:CPT:43275 | Endoscopic retrograde cholangiopancreatography (ERCP); with removal of foreign body(s) or stent(s) from biliary/pancreatic duct(s) |
|  |  |  | procedure | UMLS:CPT:43276 | Endoscopic retrograde cholangiopancreatography (ERCP); with removal and exchange of stent(s), biliary or pancreatic duct, including pre- and post-dilation and guide wire passage, when performed, including sphincterotomy, when performed, each stent exchanged |
|  |  |  | procedure | UMLS:CPT:43277 | Endoscopic retrograde cholangiopancreatography (ERCP); with trans-endoscopic balloon dilation of biliary/pancreatic duct(s) or of ampulla (sphincteroplasty), including sphincterotomy, when performed, each duct |
|  |  |  | procedure | UMLS:CPT:43278 | Endoscopic retrograde cholangiopancreatography (ERCP); with ablation of tumor(s), polyp(s), or other lesion(s), including pre- and post-dilation and guide wire passage, when performed |
|  |  | and | laboratory | TNX:9020 | Platelets [#/volume] in Blood (between 30.00 and 100.00 10*3/uL; at least 18 years old at event) |

The index event for Cohort 2 (query name: ERCP-Group B) was defined as the following:

|  | | | | | |
| --- | --- | --- | --- | --- | --- |
| Group 1 | | | | | |
|  | **Group 1A** | | | | |
|  | must have | any of | diagnosis | UMLS:ICD10CM:K80.50 | Calculus of bile duct without cholangitis or cholecystitis without obstruction |
|  |  |  | diagnosis | UMLS:ICD10CM:K80.5 | Calculus of bile duct without cholangitis or cholecystitis |
|  |  |  | diagnosis | UMLS:ICD10CM:K83.0 | Cholangitis |
|  |  |  | diagnosis | UMLS:ICD10CM:K80.4 | Calculus of bile duct with cholecystitis |
|  |  |  | diagnosis | UMLS:ICD10CM:K80.51 | Calculus of bile duct without cholangitis or cholecystitis with obstruction |
|  |  |  | diagnosis | UMLS:ICD10CM:K80.42 | Calculus of bile duct with acute cholecystitis without obstruction |
|  |  |  | diagnosis | UMLS:ICD10CM:K80.44 | Calculus of bile duct with chronic cholecystitis without obstruction |
|  |  |  | diagnosis | UMLS:ICD10CM:K80.40 | Calculus of bile duct with cholecystitis, unspecified, without obstruction |
|  |  |  | diagnosis | UMLS:ICD10CM:K80.43 | Calculus of bile duct with acute cholecystitis with obstruction |
|  |  |  | diagnosis | UMLS:ICD10CM:K80.45 | Calculus of bile duct with chronic cholecystitis with obstruction |
|  |  |  | diagnosis | UMLS:ICD10CM:K80.41 | Calculus of bile duct with cholecystitis, unspecified, with obstruction |
|  |  |  | diagnosis | UMLS:ICD10CM:K80.70 | Calculus of gallbladder and bile duct without cholecystitis without obstruction |
|  |  |  | diagnosis | UMLS:ICD10CM:K80.71 | Calculus of gallbladder and bile duct without cholecystitis with obstruction |
|  |  |  | diagnosis | UMLS:ICD10CM:K80.62 | Calculus of gallbladder and bile duct with acute cholecystitis without obstruction |
|  |  |  | diagnosis | UMLS:ICD10CM:K80.64 | Calculus of gallbladder and bile duct with chronic cholecystitis without obstruction |
|  |  |  | diagnosis | UMLS:ICD10CM:K80.63 | Calculus of gallbladder and bile duct with acute cholecystitis with obstruction |
|  |  |  | diagnosis | UMLS:ICD10CM:K80.66 | Calculus of gallbladder and bile duct with acute and chronic cholecystitis without obstruction |
|  |  |  | diagnosis | UMLS:ICD10CM:K80.67 | Calculus of gallbladder and bile duct with acute and chronic cholecystitis with obstruction |
|  |  |  | diagnosis | UMLS:ICD10CM:K80.65 | Calculus of gallbladder and bile duct with chronic cholecystitis with obstruction |
|  | date constraint | | The terms in this group occurred at any time | | |
|  | event relationship | | Any instance of Group 1B occurred within 1 day before or up to 7 days after any instance of Group 1A | | |
|  | **Group 1B** | | | | |
|  | must have | any of | procedure | UMLS:CPT:43264 | Endoscopic retrograde cholangiopancreatography (ERCP); with removal of calculi/debris from biliary/pancreatic duct(s) |
|  |  |  | procedure | UMLS:CPT:43274 | Endoscopic retrograde cholangiopancreatography (ERCP); with placement of endoscopic stent into biliary or pancreatic duct, including pre- and post-dilation and guide wire passage, when performed, including sphincterotomy, when performed, each stent |
|  |  |  | procedure | UMLS:CPT:43273 | Endoscopic cannulation of papilla with direct visualization of pancreatic/common bile duct(s) (List separately in addition to code(s) for primary procedure) |
|  |  |  | procedure | UMLS:CPT:43260 | Endoscopic retrograde cholangiopancreatography (ERCP); diagnostic, including collection of specimen(s) by brushing or washing, when performed (separate procedure) |
|  |  |  | procedure | UMLS:CPT:43261 | Endoscopic retrograde cholangiopancreatography (ERCP); with biopsy, single or multiple |
|  |  |  | procedure | UMLS:CPT:43262 | Endoscopic retrograde cholangiopancreatography (ERCP); with sphincterotomy/papillotomy |
|  |  |  | procedure | UMLS:CPT:43263 | Endoscopic retrograde cholangiopancreatography (ERCP); with pressure measurement of sphincter of Oddi |
|  |  |  | procedure | UMLS:CPT:43264 | Endoscopic retrograde cholangiopancreatography (ERCP); with removal of calculi/debris from biliary/pancreatic duct(s) |
|  |  |  | procedure | UMLS:CPT:43265 | Endoscopic retrograde cholangiopancreatography (ERCP); with destruction of calculi, any method (eg, mechanical, electrohydraulic, lithotripsy) |
|  |  |  | procedure | UMLS:CPT:43273 | Endoscopic cannulation of papilla with direct visualization of pancreatic/common bile duct(s) (List separately in addition to code(s) for primary procedure) |
|  |  |  | procedure | UMLS:CPT:43274 | Endoscopic retrograde cholangiopancreatography (ERCP); with placement of endoscopic stent into biliary or pancreatic duct, including pre- and post-dilation and guide wire passage, when performed, including sphincterotomy, when performed, each stent |
|  |  |  | procedure | UMLS:CPT:43275 | Endoscopic retrograde cholangiopancreatography (ERCP); with removal of foreign body(s) or stent(s) from biliary/pancreatic duct(s) |
|  |  |  | procedure | UMLS:CPT:43276 | Endoscopic retrograde cholangiopancreatography (ERCP); with removal and exchange of stent(s), biliary or pancreatic duct, including pre- and post-dilation and guide wire passage, when performed, including sphincterotomy, when performed, each stent exchanged |
|  |  |  | procedure | UMLS:CPT:43277 | Endoscopic retrograde cholangiopancreatography (ERCP); with trans-endoscopic balloon dilation of biliary/pancreatic duct(s) or of ampulla (sphincteroplasty), including sphincterotomy, when performed, each duct |
|  |  |  | procedure | UMLS:CPT:43278 | Endoscopic retrograde cholangiopancreatography (ERCP); with ablation of tumor(s), polyp(s), or other lesion(s), including pre- and post-dilation and guide wire passage, when performed |
|  |  | and | laboratory | TNX:9020 | Platelets [#/volume] in Blood (at least 100.00 10*3/uL; at least 18 years old at event) |

### Analyses Specifications

The Compare Outcomes Analytic supports four types of analyses: Measure of Association, Survival, Number of Instances, and Lab result distribution. The first three analyses support the “exclude patients with outcomes prior to the window” setting. This option can exclude patients from the analysis if they are not at risk for an outcome (e.g., if the outcome is a chronic disease). When "exclude patients with the outcome prior to the time window" is not checked, all patients in the cohort are included in the analysis, regardless of whether they had the outcome prior to the time window. When "exclude patients with the outcome prior to the time window" is checked, patients are excluded from the analysis if their record includes the outcome prior to the beginning of the time window. This selection will exclude all patients with the outcome prior to the index event. If the start of the time window for the analysis falls some days after the index event, patients will also be excluded if they have the outcome between the index event and the start of the time window.

### Measure of Association Analysis

The Measure of Association Analysis calculates and compares the fraction of patients with the selected outcome. The output summary includes: Patients in each Cohort (count of patients meeting query criteria); Patients with Outcome in each Cohort (of the patients in the cohort, count of patients that had the outcome in the time window); and Risk (the fraction of patients in the cohort that have the outcome in the time window, i.e. Patients with Outcome / Patients in Cohort). In addition, Risk Difference (the difference in the risks in Cohort 1 and Cohort 2), Risk Ratio (the ratio of the risks in Cohort 1 and Cohort 2), and Odds Ratio (the ratio of the odds in Cohort 1 and Cohort 2). The bar chart shows the risk of the outcome for the both cohorts.

### Survival Analysis

The Kaplan-Meier Analysis estimates probability of the outcome at a respective time interval (daily time interval is used in this analysis). In order to account for the patients who exited the cohort during the analysis period, and therefore should not be included in the analysis, censoring is applied. In this analysis, patients are removed from the analysis (censored) after the last fact in their record.

The output summary includes: Patients in each Cohort (count of patients meeting query criteria); Patients with Outcome (of the patients in the cohort, count of patients that had the outcome in the time window); Median Survival (the number of days when the survival drops below 50%; the “-” indicates that survival does not drop below 50% during the time window); and Survival Probability at End of Time Window (the % survival at the end of the time window). In addition, Log-Rank test, Hazard Ratio and test for Proportionality.

### Number of Instances Analysis

The Number of Instances Analysis calculates how many times the outcome occurred in the time window. This analysis includes two additional settings: include patients with zero instances; the definition of an instance.

Selecting to exclude patients with zero instances will remove these patients from the calculations for mean number of instances, standard deviation, or median. The histogram showing the distribution of patients by number of instances will not contain a bar for zero. Alternatively, by selecting to include patients with zero instances, the mean, standard deviation, and median for number of instances will reflect these patients. The histogram will contain a bar for zero patients.

The definition of an instance affects how counts are analyzed. By selecting Date, each calendar date on which any of the terms selected in the outcome are recorded will represent one instance. For example, if the outcome is “Med A or Med B,” and a patient has “Med A” on January 3, then both medications on January 4, then “Med B” on January 6, then that patient is considered to have three instances– January 3, January 4, and January 6. Note that if an outcome occurs across several dates (e.g. Visit: inpatient encounter), then only the start date is tracked for the purpose of counting instances. A patient who begins at stay on January 1, ends that stay on January 3, begins another stay on January 10, and ends that stay on January 15, is considered to have two instances of the outcome.

Selecting Visit as an Instance will count any visit that includes the outcome as one instance, regardless of how many times it occurred. For instance, consider a patient administered an analgesic on each of the three days that make up an inpatient stay following some index event. If analgesic is an outcome, these three administrations will represent only one instance, because all three are associated with the same visit.

The output summary includes: Patients in Cohort (count of patients meeting query criteria); Patients with Outcome (of the patients in the cohort, count of patients that had the outcome in the time window); Mean (mean of the counts); Standard Deviation (standard deviation of the counts); Median (median of the counts); and Median (1+ instances) when patients with zero instances included in the analysis. In addition, T-Test statistics testing for the difference between the cohorts is included.

### Laboratory Results Analysis

Lab Results can be included in the analysis only for the outcomes that are labs. Only the most recent lab values in the time window are included. For the lab results that are numeric, the outcome summary includes: Patients in Cohort (count of patients meeting query criteria); Patients with Outcome (of the patients in the cohort, count of patients that had the outcome in the time window); Mean (mean of the counts); and Standard Deviation (the standard deviation for lab values across patients in the cohort). In addition, T-Test statistics testing for the difference between the cohorts is included.

For the non-numeric lab results, three values are reported: counts of Negative; Positives; and Unknowns.

The counts are represented in the bar chart as percentages of the total counts.

### Outcome Definitions

Table below outlines the definitions for each outcome and the analysis specifications. For outcome definitions consisting of more than one term, at least one term must match. Please see Appendix C for the text representation of the outcome definitions.

| Sepsis | | | | |
| --- | --- | --- | --- | --- |
|  | **Outcome definition** | | | |
|  | | Diagnosis | UMLS:ICD10CM:A41 | Other sepsis |
|  | **Settings for the performed analyses** | | | |
|  | | Risk analysis | | including patients with outcome prior to the time window |
| AKI | | | | |
|  | **Outcome definition** | | | |
|  | | Diagnosis | UMLS:ICD10CM:N17 | Acute kidney failure |
|  | | Diagnosis | UMLS:ICD10CM:N17.0 | Acute kidney failure with tubular necrosis |
|  | | Diagnosis | UMLS:ICD10CM:N17.9 | Acute kidney failure, unspecified |
|  | | Diagnosis | UMLS:ICD10CM:N17.8 | Other acute kidney failure |
|  | | Diagnosis | UMLS:ICD10CM:N17.1 | Acute kidney failure with acute cortical necrosis |
|  | | Diagnosis | UMLS:ICD10CM:N17.2 | Acute kidney failure with medullary necrosis |
|  | **Settings for the performed analyses** | | | |
|  | | Risk analysis | | including patients with outcome prior to the time window |
| SBP | | | | |
|  | **Outcome definition** | | | |
|  | | Diagnosis | UMLS:ICD10CM:K65.2 | Spontaneous bacterial peritonitis |
|  | **Settings for the performed analyses** | | | |
|  | | Risk analysis | | excluding patients with outcome prior to the time window |
| All-cause mortality | | | | |
|  | **Outcome definition** | | | |
|  | | Diagnosis | UMLS:ICD10CM:R99 | Ill-defined and unknown cause of mortality |
|  | | Diagnosis | UMLS:ICD10CM:R99-R99 | Ill-defined and unknown cause of mortality (R99) |
|  | | Demographics | Deceased | Deceased |
|  | **Settings for the performed analyses** | | | |
|  | | Kaplan - Meier survival analysis | | excluding patients with outcome prior to the time window |
|  | | Risk analysis | | including patients with outcome prior to the time window |
| Jaundice | | | | |
|  | **Outcome definition** | | | |
|  | | Diagnosis | UMLS:ICD10CM:R17 | Unspecified jaundice |
|  | **Settings for the performed analyses** | | | |
|  | | Risk analysis | | including patients with outcome prior to the time window |
| Septic shock | | | | |
|  | **Outcome definition** | | | |
|  | | Diagnosis | UMLS:ICD10CM:R65.21 | Severe sepsis with septic shock |
|  | | Diagnosis | UMLS:ICD10CM:A41 | Other sepsis |
|  | | Diagnosis | UMLS:ICD10CM:T81.12 | Postprocedural septic shock |
|  | | Diagnosis | UMLS:ICD10CM:T81.12XA | Postprocedural septic shock, initial encounter |
|  | | Diagnosis | UMLS:ICD10CM:T81.12XS | Postprocedural septic shock, sequela |
|  | | Diagnosis | UMLS:ICD10CM:T81.12XD | Postprocedural septic shock, subsequent encounter |
|  | | Diagnosis | UMLS:ICD10CM:A41.01 | Sepsis due to Methicillin susceptible Staphylococcus aureus |
|  | | Diagnosis | UMLS:ICD10CM:A40 | Streptococcal sepsis |
|  | **Settings for the performed analyses** | | | |
|  | | Risk analysis | | including patients with outcome prior to the time window |
| Post-ERCP bleeding | | | | |
|  | **Outcome definition** | | | |
|  | | Diagnosis | UMLS:ICD10CM:K91.840 | Postprocedural hemorrhage of a digestive system organ or structure following a digestive system procedure |
|  | | Diagnosis | UMLS:ICD10CM:R58 | Hemorrhage, not elsewhere classified |
|  | **Settings for the performed analyses** | | | |
|  | | Risk analysis | | including patients with outcome prior to the time window |
| Post-ERCP Pancreatitis | | | | |
|  | **Outcome definition** | | | |
|  | | Diagnosis | UMLS:ICD10CM:K85.1 | Biliary acute pancreatitis |
|  | | Diagnosis | UMLS:ICD10CM:K85.11 | Biliary acute pancreatitis with uninfected necrosis |
|  | | Diagnosis | UMLS:ICD10CM:K85.12 | Biliary acute pancreatitis with infected necrosis |
|  | **Settings for the performed analyses** | | | |
|  | | Risk analysis | | including patients with outcome prior to the time window |
| Need for blood products transfusion | | | | |
|  | **Outcome definition** | | | |
|  | | Procedure | UMLS:CPT:36430 | Transfusion, blood or blood components |
|  | **Settings for the performed analyses** | | | |
|  | | Risk analysis | | including patients with outcome prior to the time window |

## Propensity Score Matching

Propensity score matching was performed on 58 characteristic(s). In the Demographics category patients were matched on Age at Index, Male, Female, White, Black or African American, Other Race, Asian, American Indian or Alaska Native, Native Hawaiian or Other Pacific Islander characteristic(s). In the Diagnosis category patients were matched on Hepatic encephalopathy, Cholangitis, Severe sepsis, Disseminated intravascular coagulation [defibrination syndrome], Ascites, Biliary cirrhosis, unspecified, Alcoholic cirrhosis of liver, Other and unspecified cirrhosis of liver, Other cirrhosis of liver, Alcoholic cirrhosis of liver without ascites, Alcoholic cirrhosis of liver with ascites, Toxic liver disease with fibrosis and cirrhosis of liver, Chronic viral hepatitis, Nonalcoholic steatohepatitis (NASH) characteristic(s). In the Medication category patients were matched on DIURETICS, aspirin, heparin, enoxaparin, apixaban, warfarin, rivaroxaban, dalteparin, bivalirudin, fondaparinux, dabigatran etexilate, argatroban, eptifibatide, edoxaban, lepirudin, tirofiban, clopidogrel, ticagrelor, dipyridamole, prasugrel, cangrelor, ticlopidine, vorapaxar characteristic(s). In the Laboratory category patients were matched on Sodium [Moles/volume] in Serum, Plasma or Blood, Creatinine [Mass/volume] in Serum, Plasma or Blood, Platelets [#/volume] in Blood, Bilirubin.total [Mass/volume] in Serum, Plasma or Blood, Albumin [Mass/volume] in Serum, Plasma or Blood, Protein [Mass/volume] in Serum or Plasma, Prothrombin time (PT) in Plasma or Blood, INR in Plasma or Blood, Urea nitrogen [Mass/volume] in Serum, Plasma or Blood, Hemoglobin [Mass/volume] in Blood, Hematocrit [Volume Fraction] of Blood, Lactate [Moles/volume] in Serum, Plasma or Blood characteristic(s). Characteristics of the cohorts before and after matching are summarized in the table below.

| **Cohort 1 and cohort 2 patient count before and after propensity score matching** | | | | | | | | | | | | |
| --- | --- | --- | --- | --- | --- | --- | --- | --- | --- | --- | --- | --- |
|  | | | Cohort | | | Patient count before matching | | | | Patient count after matching | | |
|  | | | 1 - ERCP-Group A | | | 5,157 | | | | 5,097 | | |
|  | | | 2 - ERCP-Group B | | | 12,671 | | | | 5,097 | | |
| **Propensity score density function - Before and after matching (cohort 1 - purple, cohort 2 - green)** | | | | | | | | | | | | |
|  |  | | 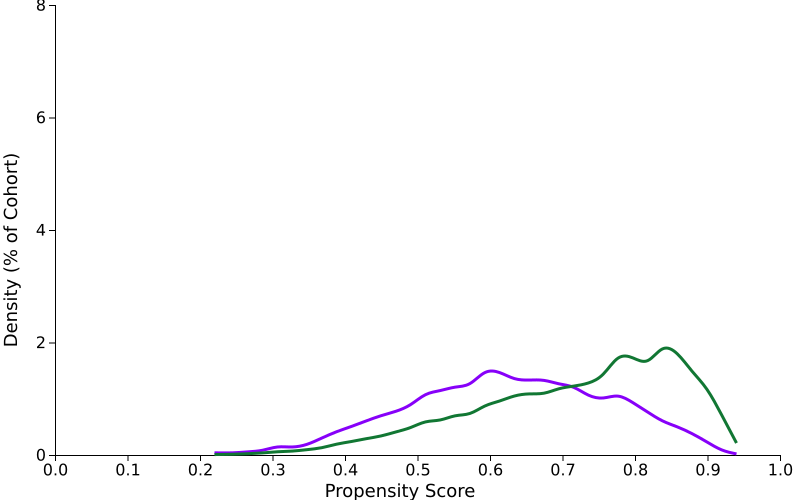 | | | | 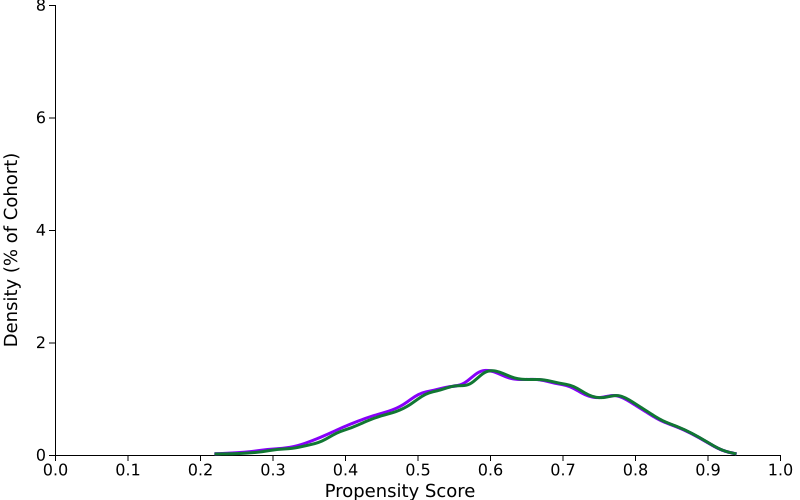 | | | | | |
| **Cohort 1 (N = 5,157) and cohort 2 (N = 12,671) characteristics before propensity score matching** | | | | | | | | | | | | |
|  | **Demographics** | | | | | | | | | | | |
|  |  | Cohort | | |  | Mean ± SD | | Patients | % of Cohort | | P-Value | Std diff. |
|  |  | 1 2 | | AI | Age at Index | 59.0 +/- 13.6 59.0 +/- 14.4 | | 5,131 12,526 | 100% 100% | | 0.727 | 0.006 |
|  |  | 1 2 | | 2106-3 | White |  | | 3,832 9,286 | 74.7% 74.1% | | 0.448 | 0.013 |
|  |  | 1 2 | | 1002-5 | American Indian or Alaska Native |  | | 39 100 | 0.8% 0.8% | | 0.794 | 0.004 |
|  |  | 1 2 | | F | Female |  | | 1,904 5,254 | 37.1% 41.9% | | <0.001 | 0.099 |
|  |  | 1 2 | | 2076-8 | Native Hawaiian or Other Pacific Islander |  | | 18 51 | 0.4% 0.4% | | 0.586 | 0.009 |
|  |  | 1 2 | | 2054-5 | Black or African American |  | | 536 1,422 | 10.4% 11.4% | | 0.082 | 0.029 |
|  |  | 1 2 | | M | Male |  | | 3,227 7,272 | 62.9% 58.1% | | <0.001 | 0.099 |
|  |  | 1 2 | | 2131-1 | Other Race |  | | 231 539 | 4.5% 4.3% | | 0.557 | 0.010 |
|  |  | 1 2 | | 2028-9 | Asian |  | | 217 525 | 4.2% 4.2% | | 0.909 | 0.002 |
|  | **Diagnosis** | | | | | | | | | | | |
|  |  | Cohort | | |  | Mean ± SD | | Patients | % of Cohort | | P-Value | Std diff. |
|  |  | 1 2 | | K76.82 | Hepatic encephalopathy |  | | 766 977 | 14.9% 7.8% | | <0.001 | 0.226 |
|  |  | 1 2 | | K83.0 | Cholangitis |  | | 2,938 5,568 | 57.3% 44.5% | | <0.001 | 0.258 |
|  |  | 1 2 | | R65.2 | Severe sepsis |  | | 1,307 1,849 | 25.5% 14.8% | | <0.001 | 0.270 |
|  |  | 1 2 | | D65 | Disseminated intravascular coagulation [defibrination syndrome] |  | | 121 149 | 2.4% 1.2% | | <0.001 | 0.089 |
|  |  | 1 2 | | R18 | Ascites |  | | 2,636 3,790 | 51.4% 30.3% | | <0.001 | 0.440 |
|  |  | 1 2 | | K74.5 | Biliary cirrhosis, unspecified |  | | 486 647 | 9.5% 5.2% | | <0.001 | 0.166 |
|  |  | 1 2 | | K70.3 | Alcoholic cirrhosis of liver |  | | 1,406 2,231 | 27.4% 17.8% | | <0.001 | 0.231 |
|  |  | 1 2 | | K74.6 | Other and unspecified cirrhosis of liver |  | | 3,539 5,768 | 69.0% 46.0% | | <0.001 | 0.477 |
|  |  | 1 2 | | K74.69 | Other cirrhosis of liver |  | | 2,365 3,392 | 46.1% 27.1% | | <0.001 | 0.403 |
|  |  | 1 2 | | K70.30 | Alcoholic cirrhosis of liver without ascites |  | | 1,199 1,892 | 23.4% 15.1% | | <0.001 | 0.211 |
|  |  | 1 2 | | K70.31 | Alcoholic cirrhosis of liver with ascites |  | | 837 1,282 | 16.3% 10.2% | | <0.001 | 0.180 |
|  |  | 1 2 | | K71.7 | Toxic liver disease with fibrosis and cirrhosis of liver |  | | 26 37 | 0.5% 0.3% | | 0.032 | 0.033 |
|  |  | 1 2 | | B18 | Chronic viral hepatitis |  | | 834 1,426 | 16.3% 11.4% | | <0.001 | 0.141 |
|  |  | 1 2 | | K75.81 | Nonalcoholic steatohepatitis (NASH) |  | | 721 1,167 | 14.1% 9.3% | | <0.001 | 0.148 |
|  | **Medication** | | | | | | | | | | | |
|  |  | Cohort | | |  | Mean ± SD | | Patients | % of Cohort | | P-Value | Std diff. |
|  |  | 1 2 | | C03 | DIURETICS |  | | 3,423 6,448 | 66.7% 51.5% | | <0.001 | 0.314 |
|  |  | 1 2 | | 1191 | aspirin |  | | 2,023 4,720 | 39.4% 37.7% | | 0.030 | 0.036 |
|  |  | 1 2 | | 5224 | heparin |  | | 3,090 6,080 | 60.2% 48.5% | | <0.001 | 0.236 |
|  |  | 1 2 | | 67108 | enoxaparin |  | | 2,160 4,584 | 42.1% 36.6% | | <0.001 | 0.113 |
|  |  | 1 2 | | 1364430 | apixaban |  | | 496 1,021 | 9.7% 8.2% | | 0.001 | 0.053 |
|  |  | 1 2 | | 11289 | warfarin |  | | 479 1,053 | 9.3% 8.4% | | 0.046 | 0.033 |
|  |  | 1 2 | | 1114195 | rivaroxaban |  | | 187 424 | 3.6% 3.4% | | 0.392 | 0.014 |
|  |  | 1 2 | | 67109 | dalteparin |  | | 109 198 | 2.1% 1.6% | | 0.012 | 0.040 |
|  |  | 1 2 | | 60819 | bivalirudin |  | | 101 219 | 2.0% 1.7% | | 0.320 | 0.016 |
|  |  | 1 2 | | 321208 | fondaparinux |  | | 48 71 | 0.9% 0.6% | | 0.007 | 0.043 |
|  |  | 1 2 | | 1037042 | dabigatran etexilate |  | | 29 60 | 0.6% 0.5% | | 0.463 | 0.012 |
|  |  | 1 2 | | 15202 | argatroban |  | | 15 20 | 0.3% 0.2% | | 0.072 | 0.028 |
|  |  | 1 2 | | 75635 | eptifibatide |  | | 10 28 | 0.2% 0.2% | | 0.709 | 0.006 |
|  |  | 1 2 | | 1599538 | edoxaban |  | | 10 10 | 0.2% 0.1% | | 0.039 | 0.031 |
|  |  | 1 2 | | 237057 | lepirudin |  | | 10 10 | 0.2% 0.1% | | 0.039 | 0.031 |
|  |  | 1 2 | | 73137 | tirofiban |  | | 0 10 | 0% 0.1% | | 0.043 | 0.040 |
|  |  | 1 2 | | 32968 | clopidogrel |  | | 350 878 | 6.8% 7.0% | | 0.655 | 0.007 |
|  |  | 1 2 | | 1116632 | ticagrelor |  | | 37 85 | 0.7% 0.7% | | 0.757 | 0.005 |
|  |  | 1 2 | | 3521 | dipyridamole |  | | 24 51 | 0.5% 0.4% | | 0.574 | 0.009 |
|  |  | 1 2 | | 613391 | prasugrel |  | | 10 37 | 0.2% 0.3% | | 0.239 | 0.020 |
|  |  | 1 2 | | 1656052 | cangrelor |  | | 10 11 | 0.2% 0.1% | | 0.061 | 0.029 |
|  |  | 1 2 | | 10594 | ticlopidine |  | | 0 10 | 0% 0.1% | | 0.043 | 0.040 |
|  |  | 1 2 | | 1537034 | vorapaxar |  | | 0 10 | 0% 0.1% | | 0.043 | 0.040 |
|  | **Laboratory** | | | | | | | | | | | |
|  |  | Cohort | | |  | Mean ± SD | | Patients | % of Cohort | | P-Value | Std diff. |
|  |  | 1 2 | | 9029 | Sodium [Moles/volume] in Serum, Plasma or Blood | 136.6 +/- 4.3 136.9 +/- 4.1 | | 4,990 11,728 | 97.3% 93.6% | | <0.001 | 0.084 |
|  |  | 1 2 | |  | 0 - 0 mmol/L |  | | 4,990 11,728 | 97.3% 93.6% | | <0.001 | 0.174 |
|  |  | 1 2 | | 9024 | Creatinine [Mass/volume] in Serum, Plasma or Blood | 1.3 +/- 2.8 1.2 +/- 2.8 | | 4,933 11,644 | 96.1% 93.0% | | 0.003 | 0.050 |
|  |  | 1 2 | |  | 0 - 0 mg/dL |  | | 4,934 11,645 | 96.2% 93.0% | | <0.001 | 0.141 |
|  |  | 1 2 | | 9020 | Platelets [#/volume] in Blood | 116.5 +/- 69.8 212.1 +/- 110.3 | | 5,001 11,758 | 97.5% 93.9% | | <0.001 | 1.034 |
|  |  | 1 2 | |  | 0 - 0 10*3/uL |  | | 5,001 11,762 | 97.5% 93.9% | | <0.001 | 0.176 |
|  |  | 1 2 | | 9044 | Alanine aminotransferase [Enzymatic activity/volume] in Serum, Plasma or Blood | 84.7 +/- 117.1 115.8 +/- 159.6 | | 4,994 11,763 | 97.3% 93.9% | | <0.001 | 0.222 |
|  |  | 1 2 | |  | 0 - 0 U/L |  | | 4,994 11,763 | 97.3% 93.9% | | <0.001 | 0.168 |
|  |  | 1 2 | | 9047 | Aspartate aminotransferase [Enzymatic activity/volume] in Serum or Plasma | 100.4 +/- 142.2 111.1 +/- 159.6 | | 4,982 11,724 | 97.1% 93.6% | | <0.001 | 0.071 |
|  |  | 1 2 | |  | 0 - 0 U/L |  | | 4,982 11,724 | 97.1% 93.6% | | <0.001 | 0.167 |
|  |  | 1 2 | | 9050 | Bilirubin.total [Mass/volume] in Serum, Plasma or Blood | 5.1 +/- 6.8 4.2 +/- 5.6 | | 4,957 11,683 | 96.6% 93.3% | | <0.001 | 0.147 |
|  |  | 1 2 | |  | 0 - 0 mg/dL |  | | 4,957 11,683 | 96.6% 93.3% | | <0.001 | 0.153 |
|  |  | 1 2 | | 9045 | Albumin [Mass/volume] in Serum, Plasma or Blood | 3.1 +/- 0.7 3.3 +/- 0.7 | | 4,888 11,503 | 95.3% 91.8% | | <0.001 | 0.326 |
|  |  | 1 2 | |  | 0 - 0 g/dL |  | | 4,888 11,503 | 95.3% 91.8% | | <0.001 | 0.140 |
|  |  | 1 2 | | 9053 | Protein [Mass/volume] in Serum or Plasma | 6.3 +/- 1.1 6.6 +/- 1.0 | | 4,912 11,510 | 95.7% 91.9% | | <0.001 | 0.286 |
|  |  | 1 2 | |  | 0 - 0 g/dL |  | | 4,912 11,510 | 95.7% 91.9% | | <0.001 | 0.160 |
|  |  | 1 2 | | 9033 | Prothrombin time (PT) in Plasma or Blood | 15.8 +/- 5.2 14.5 +/- 5.1 | | 4,697 10,276 | 91.5% 82.0% | | <0.001 | 0.256 |
|  |  | 1 2 | |  | 0 - 0 s |  | | 4,697 10,276 | 91.5% 82.0% | | <0.001 | 0.283 |
|  |  | 1 2 | | 9031 | Activated partial thromboplastin time (aPTT) in Plasma or Blood | 35.1 +/- 12.4 33.8 +/- 11.7 | | 3,934 7,939 | 76.7% 63.4% | | <0.001 | 0.114 |
|  |  | 1 2 | |  | 0 - 0 s |  | | 3,934 7,939 | 76.7% 63.4% | | <0.001 | 0.293 |
|  |  | 1 2 | | 9032 | INR in Plasma or Blood | 1.4 +/- 0.5 1.2 +/- 0.5 | | 4,821 10,581 | 94.0% 84.5% | | <0.001 | 0.265 |
|  |  | 1 2 | |  | 0 - 0 {INR} |  | | 4,821 10,581 | 94.0% 84.5% | | <0.001 | 0.309 |
|  |  | 1 2 | | 9083 | BMI | 27.2 +/- 6.1 27.8 +/- 6.7 | | 4,325 10,234 | 84.3% 81.7% | | <0.001 | 0.090 |
|  |  | 1 2 | |  | 0 - 0 kg/m2 |  | | 4,327 10,239 | 84.3% 81.7% | | <0.001 | 0.069 |
|  |  | 1 2 | | 9030 | Urea nitrogen [Mass/volume] in Serum, Plasma or Blood | 21.2 +/- 16.2 18.3 +/- 13.7 | | 4,834 11,415 | 94.2% 91.1% | | <0.001 | 0.191 |
|  |  | 1 2 | |  | 0 - 0 mg/dL |  | | 4,834 11,416 | 94.2% 91.1% | | <0.001 | 0.118 |
|  |  | 1 2 | | 9021 | Bicarbonate [Moles/volume] in Serum, Plasma or Blood | 23.4 +/- 4.0 24.1 +/- 3.7 | | 4,982 11,708 | 97.1% 93.5% | | <0.001 | 0.194 |
|  |  | 1 2 | |  | 0 - 0 mmol/L |  | | 4,983 11,711 | 97.1% 93.5% | | <0.001 | 0.172 |
|  |  | 1 2 | | 9022 | Calcium [Mass/volume] in Serum, Plasma or Blood | 8.6 +/- 0.8 8.9 +/- 0.7 | | 4,920 11,535 | 95.9% 92.1% | | <0.001 | 0.350 |
|  |  | 1 2 | |  | 0 - 0 mg/dL |  | | 4,920 11,536 | 95.9% 92.1% | | <0.001 | 0.160 |
|  |  | 1 2 | | 9015 | Leukocytes [#/volume] in Blood | 11.3 +/- 127.5 15.5 +/- 173.4 | | 4,586 10,672 | 89.4% 85.2% | | 0.145 | 0.027 |
|  |  | 1 2 | |  | 0 - 0 10*3/uL |  | | 4,587 10,688 | 89.4% 85.3% | | <0.001 | 0.123 |
|  |  | 1 2 | | 9014 | Hemoglobin [Mass/volume] in Blood | 11.0 +/- 2.3 11.8 +/- 2.3 | | 4,915 11,494 | 95.8% 91.8% | | <0.001 | 0.327 |
|  |  | 1 2 | |  | 0 - 0 g/dL |  | | 4,915 11,494 | 95.8% 91.8% | | <0.001 | 0.167 |
|  |  | 1 2 | | 9013 | Hematocrit [Volume Fraction] of Blood | 33.1 +/- 6.8 35.5 +/- 6.8 | | 4,970 11,713 | 96.9% 93.5% | | <0.001 | 0.359 |
|  |  | 1 2 | |  | 0 - 0 % |  | | 4,970 11,713 | 96.9% 93.5% | | <0.001 | 0.157 |
|  |  | 1 2 | | 9052 | Lactate dehydrogenase [Enzymatic activity/volume] in Serum or Plasma | 297.7 +/- 378.4 318.9 +/- 470.4 | | 2,299 3,925 | 44.8% 31.3% | | 0.065 | 0.050 |
|  |  | 1 2 | |  | 0 - 0 U/L |  | | 2,299 3,925 | 44.8% 31.3% | | <0.001 | 0.280 |
|  |  | 1 2 | | 9068 | Lactate [Moles/volume] in Serum, Plasma or Blood | 1.7 +/- 1.2 1.5 +/- 1.0 | | 2,730 5,266 | 53.2% 42.0% | | <0.001 | 0.144 |
|  |  | 1 2 | |  | 0 - 0 mmol/L |  | | 2,730 5,266 | 53.2% 42.0% | | <0.001 | 0.225 |
| **Cohort 1 (N = 5,097) and cohort 2 (N = 5,097) characteristics after propensity score matching** | | | | | | | | | | | | |
|  | **Demographics** | | | | | | | | | | | |
|  |  | Cohort | | |  | Mean ± SD | | Patients | % of Cohort | | P-Value | Std diff. |
|  |  | 1 2 | | AI | Age at Index | 59.0 +/- 13.6 59.4 +/- 13.5 | | 5,097 5,097 | 100% 100% | | 0.133 | 0.030 |
|  |  | 1 2 | | 2106-3 | White |  | | 3,812 3,809 | 74.8% 74.7% | | 0.945 | 0.001 |
|  |  | 1 2 | | 1002-5 | American Indian or Alaska Native |  | | 38 33 | 0.7% 0.6% | | 0.552 | 0.012 |
|  |  | 1 2 | | F | Female |  | | 1,898 1,877 | 37.2% 36.8% | | 0.667 | 0.009 |
|  |  | 1 2 | | 2076-8 | Native Hawaiian or Other Pacific Islander |  | | 18 23 | 0.4% 0.5% | | 0.434 | 0.015 |
|  |  | 1 2 | | 2054-5 | Black or African American |  | | 533 563 | 10.5% 11.0% | | 0.337 | 0.019 |
|  |  | 1 2 | | M | Male |  | | 3,199 3,220 | 62.8% 63.2% | | 0.667 | 0.009 |
|  |  | 1 2 | | 2131-1 | Other Race |  | | 225 221 | 4.4% 4.3% | | 0.846 | 0.004 |
|  |  | 1 2 | | 2028-9 | Asian |  | | 216 222 | 4.2% 4.4% | | 0.769 | 0.006 |
|  | **Diagnosis** | | | | | | | | | | | |
|  |  | Cohort | | |  | Mean ± SD | | Patients | % of Cohort | | P-Value | Std diff. |
|  |  | 1 2 | | K76.82 | Hepatic encephalopathy |  | | 752 731 | 14.8% 14.3% | | 0.555 | 0.012 |
|  |  | 1 2 | | K83.0 | Cholangitis |  | | 2,905 2,829 | 57.0% 55.5% | | 0.129 | 0.030 |
|  |  | 1 2 | | R65.2 | Severe sepsis |  | | 1,280 1,261 | 25.1% 24.7% | | 0.664 | 0.009 |
|  |  | 1 2 | | D65 | Disseminated intravascular coagulation [defibrination syndrome] |  | | 117 116 | 2.3% 2.3% | | 0.947 | 0.001 |
|  |  | 1 2 | | R18 | Ascites |  | | 2,606 2,570 | 51.1% 50.4% | | 0.476 | 0.014 |
|  |  | 1 2 | | K74.5 | Biliary cirrhosis, unspecified |  | | 470 437 | 9.2% 8.6% | | 0.251 | 0.023 |
|  |  | 1 2 | | K70.3 | Alcoholic cirrhosis of liver |  | | 1,388 1,447 | 27.2% 28.4% | | 0.192 | 0.026 |
|  |  | 1 2 | | K74.6 | Other and unspecified cirrhosis of liver |  | | 3,505 3,618 | 68.8% 71.0% | | 0.015 | 0.048 |
|  |  | 1 2 | | K74.69 | Other cirrhosis of liver |  | | 2,334 2,344 | 45.8% 46.0% | | 0.842 | 0.004 |
|  |  | 1 2 | | K70.30 | Alcoholic cirrhosis of liver without ascites |  | | 1,182 1,226 | 23.2% 24.1% | | 0.305 | 0.020 |
|  |  | 1 2 | | K70.31 | Alcoholic cirrhosis of liver with ascites |  | | 832 854 | 16.3% 16.8% | | 0.558 | 0.012 |
|  |  | 1 2 | | K71.7 | Toxic liver disease with fibrosis and cirrhosis of liver |  | | 26 22 | 0.5% 0.4% | | 0.563 | 0.011 |
|  |  | 1 2 | | B18 | Chronic viral hepatitis |  | | 823 829 | 16.1% 16.3% | | 0.872 | 0.003 |
|  |  | 1 2 | | K75.81 | Nonalcoholic steatohepatitis (NASH) |  | | 718 747 | 14.1% 14.7% | | 0.413 | 0.016 |
|  | **Medication** | | | | | | | | | | | |
|  |  | Cohort | | |  | Mean ± SD | | Patients | % of Cohort | | P-Value | Std diff. |
|  |  | 1 2 | | C03 | DIURETICS |  | | 3,392 3,380 | 66.5% 66.3% | | 0.801 | 0.005 |
|  |  | 1 2 | | 1191 | aspirin |  | | 2,017 2,121 | 39.6% 41.6% | | 0.036 | 0.042 |
|  |  | 1 2 | | 5224 | heparin |  | | 3,060 3,109 | 60.0% 61.0% | | 0.321 | 0.020 |
|  |  | 1 2 | | 67108 | enoxaparin |  | | 2,138 2,149 | 41.9% 42.2% | | 0.825 | 0.004 |
|  |  | 1 2 | | 1364430 | apixaban |  | | 492 531 | 9.7% 10.4% | | 0.199 | 0.025 |
|  |  | 1 2 | | 11289 | warfarin |  | | 476 479 | 9.3% 9.4% | | 0.919 | 0.002 |
|  |  | 1 2 | | 1114195 | rivaroxaban |  | | 185 182 | 3.6% 3.6% | | 0.873 | 0.003 |
|  |  | 1 2 | | 67109 | dalteparin |  | | 109 117 | 2.1% 2.3% | | 0.590 | 0.011 |
|  |  | 1 2 | | 60819 | bivalirudin |  | | 101 100 | 2.0% 2.0% | | 0.943 | 0.001 |
|  |  | 1 2 | | 321208 | fondaparinux |  | | 48 41 | 0.9% 0.8% | | 0.456 | 0.015 |
|  |  | 1 2 | | 1037042 | dabigatran etexilate |  | | 28 32 | 0.5% 0.6% | | 0.605 | 0.010 |
|  |  | 1 2 | | 15202 | argatroban |  | | 15 15 | 0.3% 0.3% | | 1 | <0.001 |
|  |  | 1 2 | | 75635 | eptifibatide |  | | 10 11 | 0.2% 0.2% | | 0.827 | 0.004 |
|  |  | 1 2 | | 1599538 | edoxaban |  | | 10 10 | 0.2% 0.2% | | 1 | <0.001 |
|  |  | 1 2 | | 237057 | lepirudin |  | | 10 10 | 0.2% 0.2% | | 1 | <0.001 |
|  |  | 1 2 | | 73137 | tirofiban |  | | 0 10 | 0% 0.2% | | 0.002 | 0.063 |
|  |  | 1 2 | | 32968 | clopidogrel |  | | 349 363 | 6.8% 7.1% | | 0.586 | 0.011 |
|  |  | 1 2 | | 1116632 | ticagrelor |  | | 36 35 | 0.7% 0.7% | | 0.905 | 0.002 |
|  |  | 1 2 | | 3521 | dipyridamole |  | | 24 23 | 0.5% 0.5% | | 0.884 | 0.003 |
|  |  | 1 2 | | 613391 | prasugrel |  | | 10 10 | 0.2% 0.2% | | 1 | <0.001 |
|  |  | 1 2 | | 1656052 | cangrelor |  | | 10 10 | 0.2% 0.2% | | 1 | <0.001 |
|  |  | 1 2 | | 10594 | ticlopidine |  | | 0 0 | 0% 0% | | -- | -- |
|  |  | 1 2 | | 1537034 | vorapaxar |  | | 0 0 | 0% 0% | | -- | -- |
|  | **Laboratory** | | | | | | | | | | | |
|  |  | Cohort | | |  | Mean ± SD | | Patients | % of Cohort | | P-Value | Std diff. |
|  |  | 1 2 | | 9029 | Sodium [Moles/volume] in Serum, Plasma or Blood | 136.6 +/- 4.3 136.4 +/- 4.3 | | 4,956 4,955 | 97.2% 97.2% | | 0.216 | 0.025 |
|  |  | 1 2 | |  | 0 - 0 mmol/L |  | | 4,956 4,955 | 97.2% 97.2% | | 0.952 | 0.001 |
|  |  | 1 2 | | 9024 | Creatinine [Mass/volume] in Serum, Plasma or Blood | 1.3 +/- 2.8 1.3 +/- 3.6 | | 4,899 4,893 | 96.1% 96.0% | | 0.781 | 0.006 |
|  |  | 1 2 | |  | 0 - 0 mg/dL |  | | 4,900 4,893 | 96.1% 96.0% | | 0.721 | 0.007 |
|  |  | 1 2 | | 9020 | Platelets [#/volume] in Blood | 116.5 +/- 69.5 180.8 +/- 105.5 | | 4,967 4,969 | 97.4% 97.5% | | <0.001 | 0.720 |
|  |  | 1 2 | |  | 0 - 0 10*3/uL |  | | 4,967 4,969 | 97.4% 97.5% | | 0.900 | 0.002 |
|  |  | 1 2 | | 9044 | Alanine aminotransferase [Enzymatic activity/volume] in Serum, Plasma or Blood | 84.8 +/- 117.0 97.8 +/- 126.7 | | 4,960 4,961 | 97.3% 97.3% | | <0.001 | 0.106 |
|  |  | 1 2 | |  | 0 - 0 U/L |  | | 4,960 4,961 | 97.3% 97.3% | | 0.951 | 0.001 |
|  |  | 1 2 | | 9047 | Aspartate aminotransferase [Enzymatic activity/volume] in Serum or Plasma | 100.4 +/- 142.2 104.1 +/- 143.7 | | 4,948 4,951 | 97.1% 97.1% | | 0.207 | 0.025 |
|  |  | 1 2 | |  | 0 - 0 U/L |  | | 4,948 4,951 | 97.1% 97.1% | | 0.859 | 0.004 |
|  |  | 1 2 | | 9050 | Bilirubin.total [Mass/volume] in Serum, Plasma or Blood | 5.0 +/- 6.7 4.7 +/- 6.2 | | 4,923 4,917 | 96.6% 96.5% | | 0.017 | 0.048 |
|  |  | 1 2 | |  | 0 - 0 mg/dL |  | | 4,923 4,917 | 96.6% 96.5% | | 0.745 | 0.006 |
|  |  | 1 2 | | 9045 | Albumin [Mass/volume] in Serum, Plasma or Blood | 3.1 +/- 0.7 3.2 +/- 0.8 | | 4,854 4,847 | 95.2% 95.1% | | <0.001 | 0.135 |
|  |  | 1 2 | |  | 0 - 0 g/dL |  | | 4,854 4,847 | 95.2% 95.1% | | 0.747 | 0.006 |
|  |  | 1 2 | | 9053 | Protein [Mass/volume] in Serum or Plasma | 6.3 +/- 1.1 6.5 +/- 1.0 | | 4,878 4,877 | 95.7% 95.7% | | <0.001 | 0.115 |
|  |  | 1 2 | |  | 0 - 0 g/dL |  | | 4,878 4,877 | 95.7% 95.7% | | 0.961 | 0.001 |
|  |  | 1 2 | | 9033 | Prothrombin time (PT) in Plasma or Blood | 15.8 +/- 5.2 15.0 +/- 5.0 | | 4,663 4,704 | 91.5% 92.3% | | <0.001 | 0.144 |
|  |  | 1 2 | |  | 0 - 0 s |  | | 4,663 4,704 | 91.5% 92.3% | | 0.137 | 0.029 |
|  |  | 1 2 | | 9031 | Activated partial thromboplastin time (aPTT) in Plasma or Blood | 35.1 +/- 12.3 34.5 +/- 12.2 | | 3,904 3,864 | 76.6% 75.8% | | 0.030 | 0.049 |
|  |  | 1 2 | |  | 0 - 0 s |  | | 3,904 3,864 | 76.6% 75.8% | | 0.352 | 0.018 |
|  |  | 1 2 | | 9032 | INR in Plasma or Blood | 1.4 +/- 0.5 1.3 +/- 0.4 | | 4,787 4,827 | 93.9% 94.7% | | <0.001 | 0.170 |
|  |  | 1 2 | |  | 0 - 0 {INR} |  | | 4,787 4,827 | 93.9% 94.7% | | 0.087 | 0.034 |
|  |  | 1 2 | | 9083 | BMI | 27.3 +/- 6.2 27.4 +/- 6.4 | | 4,292 4,315 | 84.2% 84.7% | | 0.171 | 0.030 |
|  |  | 1 2 | |  | 0 - 0 kg/m2 |  | | 4,294 4,316 | 84.2% 84.7% | | 0.548 | 0.012 |
|  |  | 1 2 | | 9030 | Urea nitrogen [Mass/volume] in Serum, Plasma or Blood | 21.1 +/- 16.1 20.3 +/- 15.0 | | 4,801 4,798 | 94.2% 94.1% | | 0.011 | 0.052 |
|  |  | 1 2 | |  | 0 - 0 mg/dL |  | | 4,801 4,799 | 94.2% 94.2% | | 0.933 | 0.002 |
|  |  | 1 2 | | 9021 | Bicarbonate [Moles/volume] in Serum, Plasma or Blood | 23.4 +/- 4.0 23.6 +/- 3.9 | | 4,948 4,948 | 97.1% 97.1% | | 0.007 | 0.054 |
|  |  | 1 2 | |  | 0 - 0 mmol/L |  | | 4,949 4,950 | 97.1% 97.1% | | 0.953 | 0.001 |
|  |  | 1 2 | | 9022 | Calcium [Mass/volume] in Serum, Plasma or Blood | 8.6 +/- 0.8 8.7 +/- 0.8 | | 4,886 4,876 | 95.9% 95.7% | | <0.001 | 0.191 |
|  |  | 1 2 | |  | 0 - 0 mg/dL |  | | 4,886 4,877 | 95.9% 95.7% | | 0.658 | 0.009 |
|  |  | 1 2 | | 9015 | Leukocytes [#/volume] in Blood | 11.3 +/- 127.9 14.2 +/- 160.4 | | 4,554 4,523 | 89.3% 88.7% | | 0.343 | 0.020 |
|  |  | 1 2 | |  | 0 - 0 10*3/uL |  | | 4,555 4,529 | 89.4% 88.9% | | 0.408 | 0.016 |
|  |  | 1 2 | | 9014 | Hemoglobin [Mass/volume] in Blood | 11.0 +/- 2.3 11.3 +/- 2.3 | | 4,881 4,862 | 95.8% 95.4% | | <0.001 | 0.110 |
|  |  | 1 2 | |  | 0 - 0 g/dL |  | | 4,881 4,862 | 95.8% 95.4% | | 0.360 | 0.018 |
|  |  | 1 2 | | 9013 | Hematocrit [Volume Fraction] of Blood | 33.1 +/- 6.8 34.0 +/- 6.9 | | 4,936 4,929 | 96.8% 96.7% | | <0.001 | 0.130 |
|  |  | 1 2 | |  | 0 - 0 % |  | | 4,936 4,929 | 96.8% 96.7% | | 0.695 | 0.008 |
|  |  | 1 2 | | 9052 | Lactate dehydrogenase [Enzymatic activity/volume] in Serum or Plasma | 296.0 +/- 376.1 322.7 +/- 498.0 | | 2,273 2,143 | 44.6% 42.0% | | 0.044 | 0.060 |
|  |  | 1 2 | |  | 0 - 0 U/L |  | | 2,273 2,143 | 44.6% 42.0% | | 0.009 | 0.051 |
|  |  | 1 2 | | 9068 | Lactate [Moles/volume] in Serum, Plasma or Blood | 1.7 +/- 1.2 1.6 +/- 1.1 | | 2,701 2,729 | 53.0% 53.5% | | <0.001 | 0.095 |
|  |  | 1 2 | |  | 0 - 0 mmol/L |  | | 2,701 2,729 | 53.0% 53.5% | | 0.578 | 0.011 |

# Results

Results are summarized in the tables below. Outcomes analysis was performed on the cohorts after propensity score matching.

| **1 Sepsis** | | | | | | | | | | | | |
| --- | --- | --- | --- | --- | --- | --- | --- | --- | --- | --- | --- | --- |
|  | | **Risk analysis** | | | | | | | | | | |
|  |  | | | Cohort | | | Patients in cohort | Patients with outcome | Risk | | | |
|  | | |  | 1 | | ERCP-Group A | 5,097 | 769 | 0.151 | | | |
|  | | |  | 2 | | ERCP-Group B | 5,097 | 739 | 0.145 | | | |
|  | | | | | | | | | | | | |
|  | | |  |  | | |  | 95% CI | z | p |  |  |
|  | | |  | **Risk Difference** | | | 0.006 | (-0.008, 0.020) | 0.837 | 0.403 |  |  |
|  | | |  | **Risk Ratio** | | | 1.041 | (0.948, 1.142) | N/A | N/A |  |  |
|  | | |  | **Odds Ratio** | | | 1.048 | (0.939, 1.169) | N/A | N/A |  |  |
|  | | | | | | | | | | | | |
|  | |  | | | 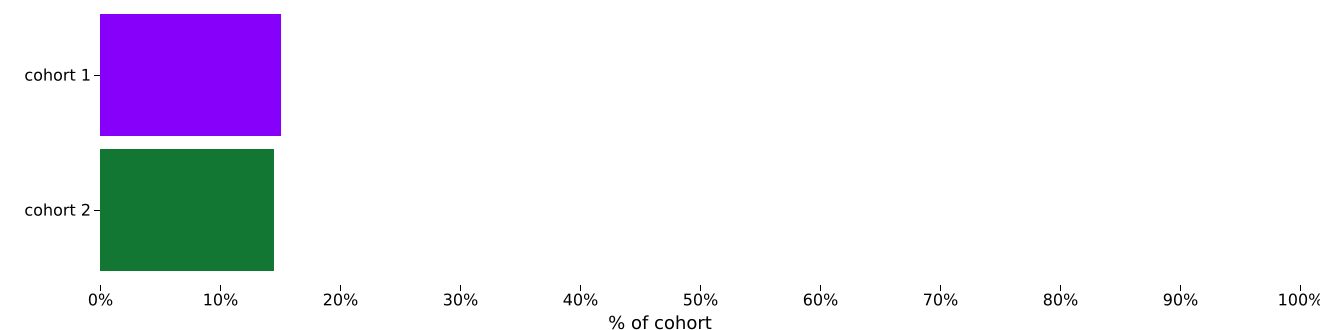 | | | | | | | |
| **2 AKI** | | | | | | | | | | | | |
|  | | **Risk analysis** | | | | | | | | | | |
|  |  | | | Cohort | | | Patients in cohort | Patients with outcome | Risk | | | |
|  | | |  | 1 | | ERCP-Group A | 5,097 | 991 | 0.194 | | | |
|  | | |  | 2 | | ERCP-Group B | 5,097 | 907 | 0.178 | | | |
|  | | | | | | | | | | | | |
|  | | |  |  | | |  | 95% CI | z | p |  |  |
|  | | |  | **Risk Difference** | | | 0.016 | (0.001, 0.032) | 2.137 | 0.033 |  |  |
|  | | |  | **Risk Ratio** | | | 1.093 | (1.007, 1.185) | N/A | N/A |  |  |
|  | | |  | **Odds Ratio** | | | 1.115 | (1.009, 1.232) | N/A | N/A |  |  |
|  | | | | | | | | | | | | |
|  | |  | | | 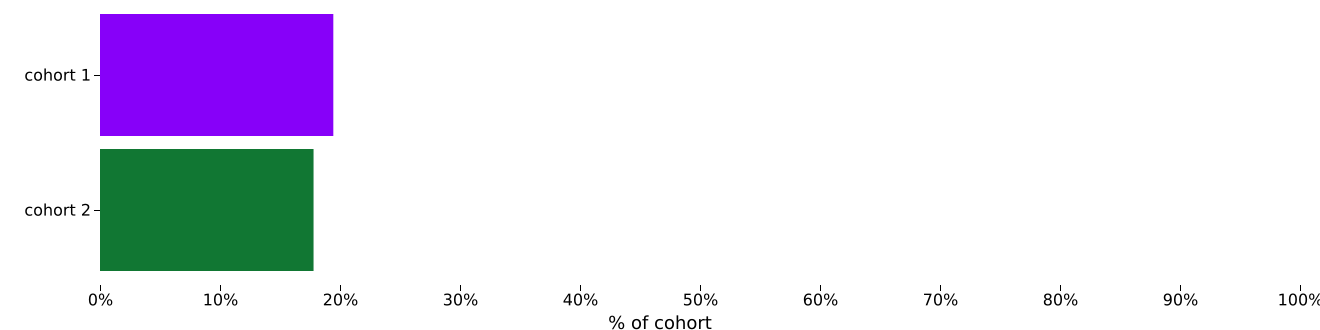 | | | | | | | |
| **3 SBP** | | | | | | | | | | | | |
|  | | **Risk analysis excluding patients with outcome prior to the time window** | | | | | | | | | | |
|  |  | | | Cohort | | | Patients in cohort | Patients with outcome | Risk | | | |
|  | | |  | 1 | | ERCP-Group A | 4,654 | 60 | 0.013 | | | |
|  | | |  | 2 | | ERCP-Group B | 4,652 | 41 | 0.009 | | | |
|  | | | | | | | | | | | | |
|  | | |  |  | | |  | 95% CI | z | p |  |  |
|  | | |  | **Risk Difference** | | | 0.004 | (-0.000, 0.008) | 1.899 | 0.058 |  |  |
|  | | |  | **Risk Ratio** | | | 1.463 | (0.985, 2.171) | N/A | N/A |  |  |
|  | | |  | **Odds Ratio** | | | 1.469 | (0.985, 2.190) | N/A | N/A |  |  |
|  | | | | | | | | | | | | |
|  | |  | | | 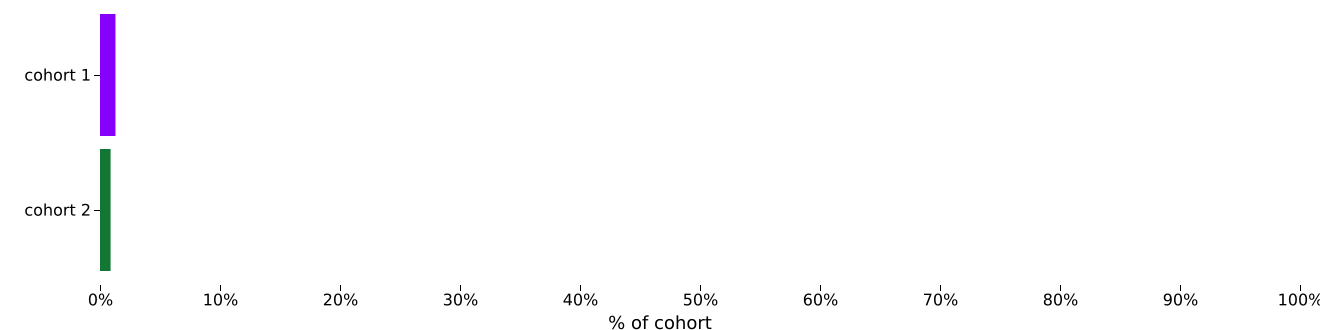 | | | | | | | |
|  | |  | | | 443 patients in Cohort 1 and 445 patients in Cohort 2 were excluded from results because they had the outcome prior to the time window. | | | | | | | |
| **4 All-cause mortality** | | | | | | | | | | | | |
|  | | **Risk analysis** | | | | | | | | | | |
|  |  | | | Cohort | | | Patients in cohort | Patients with outcome | Risk | | | |
|  | | |  | 1 | | ERCP-Group A | 5,097 | 440 | 0.086 | | | |
|  | | |  | 2 | | ERCP-Group B | 5,097 | 294 | 0.058 | | | |
|  | | | | | | | | | | | | |
|  | | |  |  | | |  | 95% CI | z | p |  |  |
|  | | |  | **Risk Difference** | | | 0.029 | (0.019, 0.039) | 5.594 | 0.000 |  |  |
|  | | |  | **Risk Ratio** | | | 1.497 | (1.298, 1.726) | N/A | N/A |  |  |
|  | | |  | **Odds Ratio** | | | 1.544 | (1.324, 1.799) | N/A | N/A |  |  |
|  | | | | | | | | | | | | |
|  | |  | | | 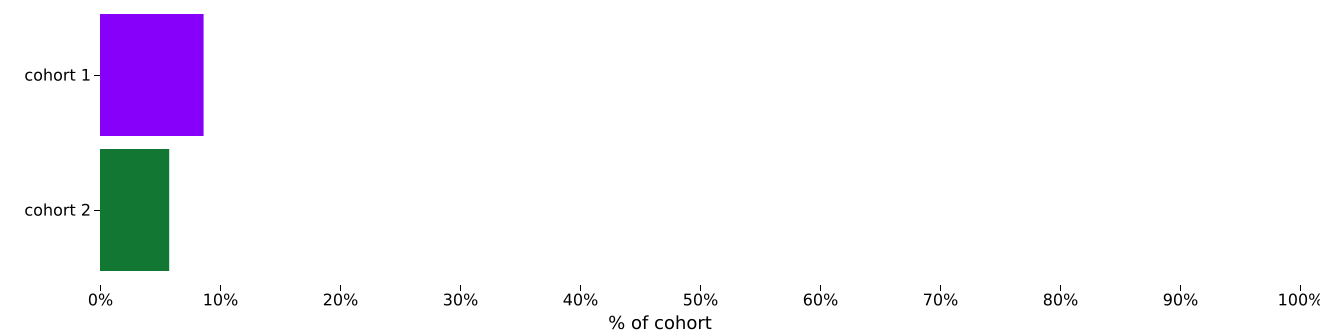 | | | | | | | |
|  | | **Kaplan - Meier survival analysis excluding patients with outcome prior to the time window** | | | | | | | | | | |
|  | | |  | Cohort | | | Patients in cohort | Patients with outcome | Median survival (days) | Survival probability at end of time window | | |
|  | | |  | 1 | | ERCP-Group A | 4,899 | 401 | -- | 91.62% | | |
|  | | |  | 2 | | ERCP-Group B | 4,910 | 269 | -- | 94.42% | | |
|  | | | | | | | | | | | | |
|  | | |  |  | | | χ^2^ | df | p |  |  |  |
|  | | |  | **Log-Rank Test** | | | 29.165 | 1 | 0.000 |  |  |  |
|  | | | | | | | | | | | | |
|  | | |  |  | | | Hazard Ratio | 95% CI | χ^2^ | df | p | |
|  | | |  | **Hazard Ratio and Proportionality** | | | 1.525 | (1.307, 1.780) | 0.692 | 1 | 0.405 | |
|  | | | | | | | | | | | | |
|  | |  | | | 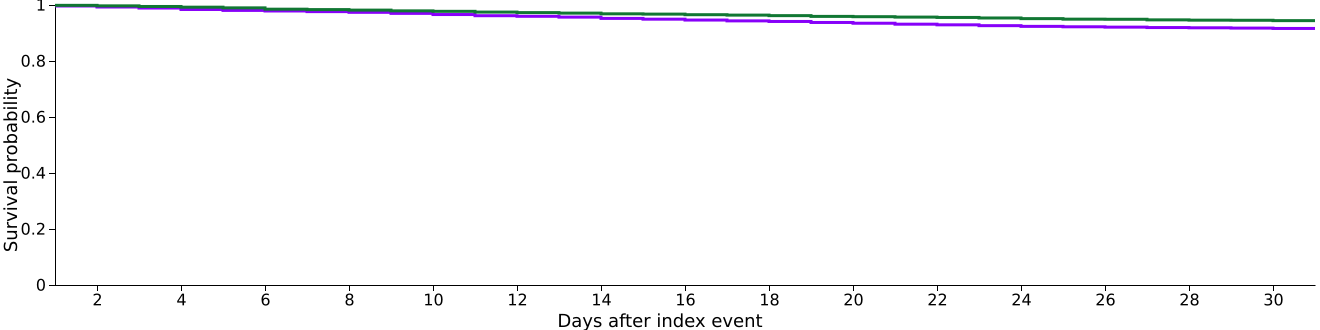 | | | | | | | |
|  | |  | | | 198 patients in Cohort 1 and 187 patients in Cohort 2 were excluded from results because they had the outcome prior to the time window. | | | | | | | |
| **5 Jaundice** | | | | | | | | | | | | |
|  | | **Risk analysis** | | | | | | | | | | |
|  |  | | | Cohort | | | Patients in cohort | Patients with outcome | Risk | | | |
|  | | |  | 1 | | ERCP-Group A | 5,097 | 487 | 0.096 | | | |
|  | | |  | 2 | | ERCP-Group B | 5,097 | 526 | 0.103 | | | |
|  | | | | | | | | | | | | |
|  | | |  |  | | |  | 95% CI | z | p |  |  |
|  | | |  | **Risk Difference** | | | -0.008 | (-0.019, 0.004) | -1.291 | 0.197 |  |  |
|  | | |  | **Risk Ratio** | | | 0.926 | (0.824, 1.041) | N/A | N/A |  |  |
|  | | |  | **Odds Ratio** | | | 0.918 | (0.806, 1.045) | N/A | N/A |  |  |
|  | | | | | | | | | | | | |
|  | |  | | | 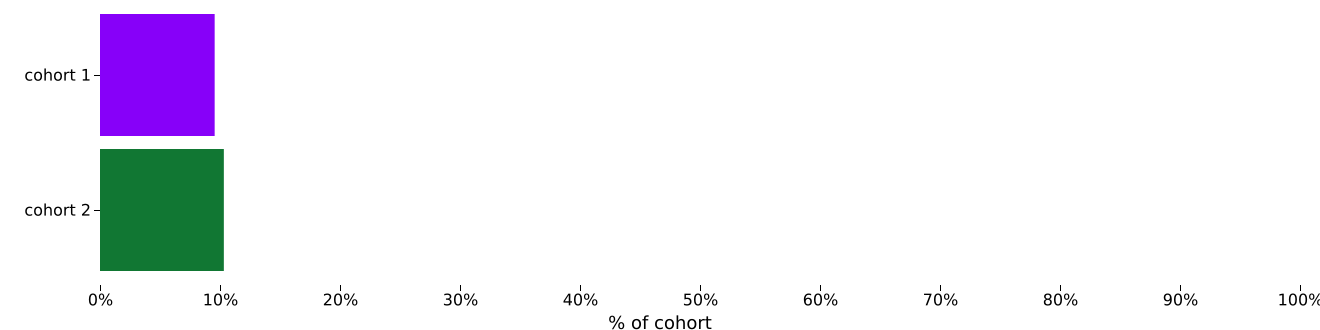 | | | | | | | |
| **6 Septic shock** | | | | | | | | | | | | |
|  | | **Risk analysis** | | | | | | | | | | |
|  |  | | | Cohort | | | Patients in cohort | Patients with outcome | Risk | | | |
|  | | |  | 1 | | ERCP-Group A | 5,097 | 793 | 0.156 | | | |
|  | | |  | 2 | | ERCP-Group B | 5,097 | 765 | 0.150 | | | |
|  | | | | | | | | | | | | |
|  | | |  |  | | |  | 95% CI | z | p |  |  |
|  | | |  | **Risk Difference** | | | 0.005 | (-0.008, 0.019) | 0.771 | 0.441 |  |  |
|  | | |  | **Risk Ratio** | | | 1.037 | (0.946, 1.136) | N/A | N/A |  |  |
|  | | |  | **Odds Ratio** | | | 1.043 | (0.937, 1.162) | N/A | N/A |  |  |
|  | | | | | | | | | | | | |
|  | |  | | | 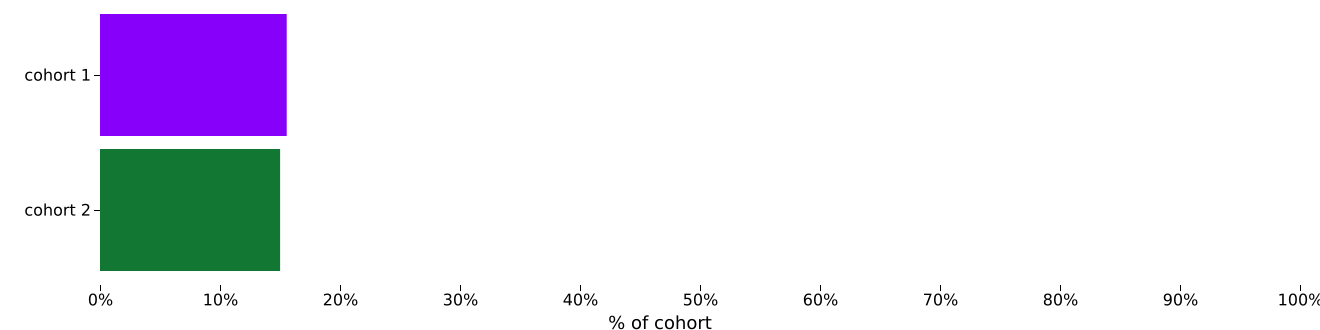 | | | | | | | |
| **7 Post-ERCP bleeding** | | | | | | | | | | | | |
|  | | **Risk analysis** | | | | | | | | | | |
|  |  | | | Cohort | | | Patients in cohort | Patients with outcome | Risk | | | |
|  | | |  | 1 | | ERCP-Group A | 5,097 | 90 | 0.018 | | | |
|  | | |  | 2 | | ERCP-Group B | 5,097 | 93 | 0.018 | | | |
|  | | | | | | | | | | | | |
|  | | |  |  | | |  | 95% CI | z | p |  |  |
|  | | |  | **Risk Difference** | | | -0.001 | (-0.006, 0.005) | -0.224 | 0.823 |  |  |
|  | | |  | **Risk Ratio** | | | 0.968 | (0.726, 1.290) | N/A | N/A |  |  |
|  | | |  | **Odds Ratio** | | | 0.967 | (0.722, 1.296) | N/A | N/A |  |  |
|  | | | | | | | | | | | | |
|  | |  | | | 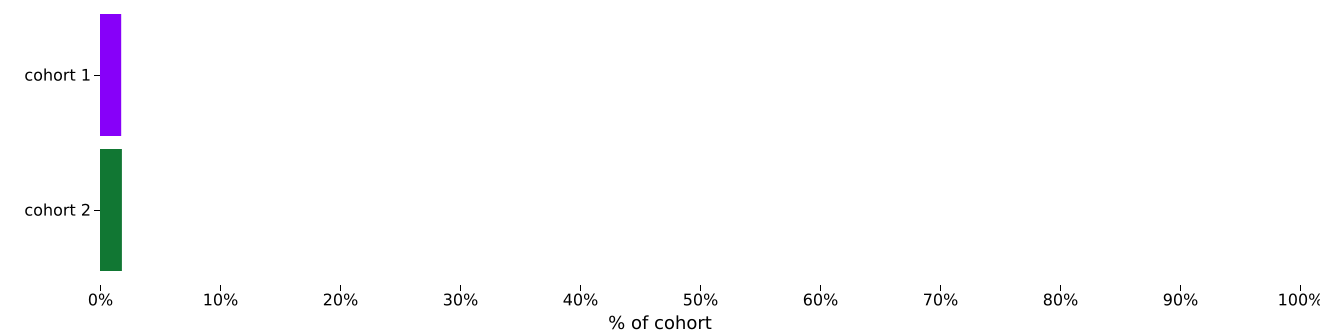 | | | | | | | |
| **8 Post-ERCP Pancreatitis** | | | | | | | | | | | | |
|  | | **Risk analysis** | | | | | | | | | | |
|  |  | | | Cohort | | | Patients in cohort | Patients with outcome | Risk | | | |
|  | | |  | 1 | | ERCP-Group A | 5,097 | 137 | 0.027 | | | |
|  | | |  | 2 | | ERCP-Group B | 5,097 | 167 | 0.033 | | | |
|  | | | | | | | | | | | | |
|  | | |  |  | | |  | 95% CI | z | p |  |  |
|  | | |  | **Risk Difference** | | | -0.006 | (-0.012, 0.001) | -1.747 | 0.081 |  |  |
|  | | |  | **Risk Ratio** | | | 0.820 | (0.657, 1.025) | N/A | N/A |  |  |
|  | | |  | **Odds Ratio** | | | 0.815 | (0.648, 1.026) | N/A | N/A |  |  |
|  | | | | | | | | | | | | |
|  | |  | | | 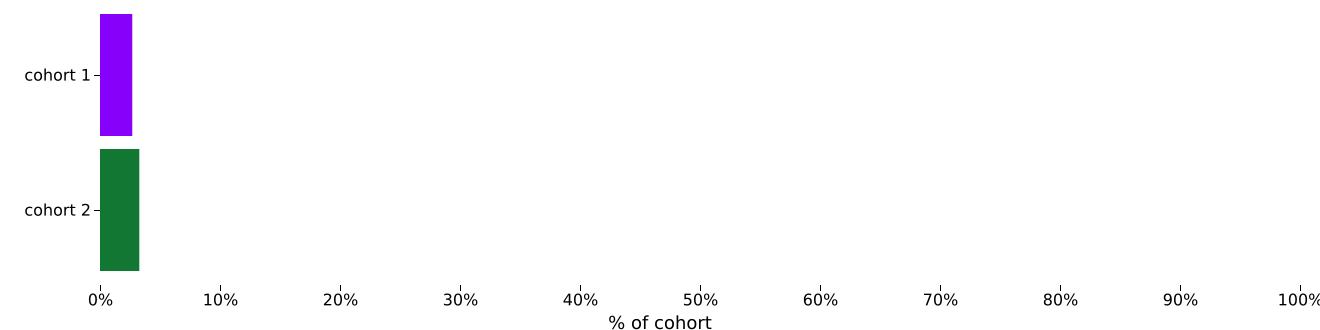 | | | | | | | |
| **9 Need for blood products transfusion** | | | | | | | | | | | | |
|  | | **Risk analysis** | | | | | | | | | | |
|  |  | | | Cohort | | | Patients in cohort | Patients with outcome | Risk | | | |
|  | | |  | 1 | | ERCP-Group A | 5,097 | 369 | 0.072 | | | |
|  | | |  | 2 | | ERCP-Group B | 5,097 | 274 | 0.054 | | | |
|  | | | | | | | | | | | | |
|  | | |  |  | | |  | 95% CI | z | p |  |  |
|  | | |  | **Risk Difference** | | | 0.019 | (0.009, 0.028) | 3.870 | 0.000 |  |  |
|  | | |  | **Risk Ratio** | | | 1.347 | (1.157, 1.567) | N/A | N/A |  |  |
|  | | |  | **Odds Ratio** | | | 1.374 | (1.169, 1.614) | N/A | N/A |  |  |
|  | | | | | | | | | | | | |
|  | |  | | | 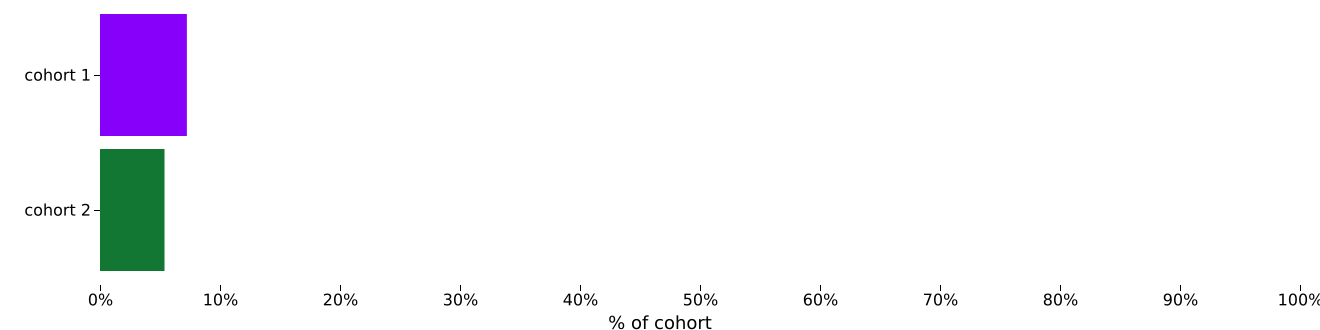 | | | | | | | |

# Appendix A – Text Representation of the Cohorts Definition

This section lists all terms used in the definitions of the two cohorts.

### Query Criteria for Cohort 1 (query name: ERCP-Group A)

Patients must have:
 all of the following:
 Age (Age) (between 18 and 89 years (most recent occurrence)); and
 any of the following:
 Unspecified cirrhosis of liver (UMLS:ICD10CM:K74.60); or
 Biliary cirrhosis, unspecified (UMLS:ICD10CM:K74.5); or
 Toxic liver disease with fibrosis and cirrhosis of liver (UMLS:ICD10CM:K71.7); or
 Primary biliary cirrhosis (UMLS:ICD10CM:K74.3); or
 Secondary biliary cirrhosis (UMLS:ICD10CM:K74.4); or
 Other and unspecified cirrhosis of liver (UMLS:ICD10CM:K74.6); or
 Alcoholic hepatitis with ascites (UMLS:ICD10CM:K70.11); or
 Chronic passive congestion of liver (UMLS:ICD10CM:K76.1); or
 Alcoholic cirrhosis of liver without ascites (UMLS:ICD10CM:K70.30); or
 Alcoholic cirrhosis of liver without ascites (UMLS:ICD10CM:K70.30); or
 Primary biliary cirrhosis (UMLS:ICD10CM:K74.3); or
 Alcoholic cirrhosis of liver with ascites (UMLS:ICD10CM:K70.31); or
 Hepatic fibrosis, unspecified (UMLS:ICD10CM:K74.00).

All the following must be satisfied:

 Group 1A: The terms in this group occurred at any time
 Patients must have:
 any of the following:
 Calculus of bile duct without cholangitis or cholecystitis without obstruction (UMLS:ICD10CM:K80.50); or
 Calculus of bile duct without cholangitis or cholecystitis (UMLS:ICD10CM:K80.5); or
 Cholangitis (UMLS:ICD10CM:K83.0); or
 Calculus of bile duct with cholecystitis (UMLS:ICD10CM:K80.4); or
 Calculus of bile duct without cholangitis or cholecystitis with obstruction (UMLS:ICD10CM:K80.51); or
 Calculus of bile duct with acute cholecystitis without obstruction (UMLS:ICD10CM:K80.42); or
 Calculus of bile duct with chronic cholecystitis without obstruction (UMLS:ICD10CM:K80.44); or
 Calculus of bile duct with cholecystitis, unspecified, without obstruction (UMLS:ICD10CM:K80.40); or
 Calculus of bile duct with acute cholecystitis with obstruction (UMLS:ICD10CM:K80.43); or
 Calculus of bile duct with chronic cholecystitis with obstruction (UMLS:ICD10CM:K80.45); or
 Calculus of bile duct with cholecystitis, unspecified, with obstruction (UMLS:ICD10CM:K80.41); or
 Calculus of gallbladder and bile duct without cholecystitis without obstruction (UMLS:ICD10CM:K80.70); or
 Calculus of gallbladder and bile duct without cholecystitis with obstruction (UMLS:ICD10CM:K80.71); or
 Calculus of gallbladder and bile duct with acute cholecystitis without obstruction (UMLS:ICD10CM:K80.62); or
 Calculus of gallbladder and bile duct with chronic cholecystitis without obstruction (UMLS:ICD10CM:K80.64); or
 Calculus of gallbladder and bile duct with acute cholecystitis with obstruction (UMLS:ICD10CM:K80.63); or
 Calculus of gallbladder and bile duct with acute and chronic cholecystitis without obstruction (UMLS:ICD10CM:K80.66); or
 Calculus of gallbladder and bile duct with acute and chronic cholecystitis with obstruction (UMLS:ICD10CM:K80.67); or
 Calculus of gallbladder and bile duct with chronic cholecystitis with obstruction (UMLS:ICD10CM:K80.65).
 Group 1B: Any instance of Group 1B occurred within 1 day before or up to 7 days after any instance of Group 1A
 Patients must have:
 all of the following:
 any of the following:
 Endoscopic retrograde cholangiopancreatography (ERCP); with removal of calculi/debris from biliary/pancreatic duct(s) (UMLS:CPT:43264); or
 Endoscopic retrograde cholangiopancreatography (ERCP); with placement of endoscopic stent into biliary or pancreatic duct, including pre- and post-dilation and guide wire passage, when performed, including sphincterotomy, when performed, each stent (UMLS:CPT:43274); or
 Endoscopic cannulation of papilla with direct visualization of pancreatic/common bile duct(s) (List separately in addition to code(s) for primary procedure) (UMLS:CPT:43273); or
 Endoscopic retrograde cholangiopancreatography (ERCP); diagnostic, including collection of specimen(s) by brushing or washing, when performed (separate procedure) (UMLS:CPT:43260); or
 Endoscopic retrograde cholangiopancreatography (ERCP); with biopsy, single or multiple (UMLS:CPT:43261); or
 Endoscopic retrograde cholangiopancreatography (ERCP); with sphincterotomy/papillotomy (UMLS:CPT:43262); or
 Endoscopic retrograde cholangiopancreatography (ERCP); with pressure measurement of sphincter of Oddi (UMLS:CPT:43263); or
 Endoscopic retrograde cholangiopancreatography (ERCP); with removal of calculi/debris from biliary/pancreatic duct(s) (UMLS:CPT:43264); or
 Endoscopic retrograde cholangiopancreatography (ERCP); with destruction of calculi, any method (eg, mechanical, electrohydraulic, lithotripsy) (UMLS:CPT:43265); or
 Endoscopic cannulation of papilla with direct visualization of pancreatic/common bile duct(s) (List separately in addition to code(s) for primary procedure) (UMLS:CPT:43273); or
 Endoscopic retrograde cholangiopancreatography (ERCP); with placement of endoscopic stent into biliary or pancreatic duct, including pre- and post-dilation and guide wire passage, when performed, including sphincterotomy, when performed, each stent (UMLS:CPT:43274); or
 Endoscopic retrograde cholangiopancreatography (ERCP); with removal of foreign body(s) or stent(s) from biliary/pancreatic duct(s) (UMLS:CPT:43275); or
 Endoscopic retrograde cholangiopancreatography (ERCP); with removal and exchange of stent(s), biliary or pancreatic duct, including pre- and post-dilation and guide wire passage, when performed, including sphincterotomy, when performed, each stent exchanged (UMLS:CPT:43276); or
 Endoscopic retrograde cholangiopancreatography (ERCP); with trans-endoscopic balloon dilation of biliary/pancreatic duct(s) or of ampulla (sphincteroplasty), including sphincterotomy, when performed, each duct (UMLS:CPT:43277); or
 Endoscopic retrograde cholangiopancreatography (ERCP); with ablation of tumor(s), polyp(s), or other lesion(s), including pre- and post-dilation and guide wire passage, when performed (UMLS:CPT:43278); and
 Platelets [#/volume] in Blood (TNX:9020) (between 30.00 and 100.00 10*3/uL; and at least 18 years old at event).

### Query Criteria for Cohort 2 (query name: ERCP-Group B)

Patients must have:
 all of the following:
 Age (Age) (between 18 and 89 years (most recent occurrence)); and
 any of the following:
 Unspecified cirrhosis of liver (UMLS:ICD10CM:K74.60); or
 Biliary cirrhosis, unspecified (UMLS:ICD10CM:K74.5); or
 Toxic liver disease with fibrosis and cirrhosis of liver (UMLS:ICD10CM:K71.7); or
 Primary biliary cirrhosis (UMLS:ICD10CM:K74.3); or
 Secondary biliary cirrhosis (UMLS:ICD10CM:K74.4); or
 Other and unspecified cirrhosis of liver (UMLS:ICD10CM:K74.6); or
 Alcoholic hepatitis with ascites (UMLS:ICD10CM:K70.11); or
 Chronic passive congestion of liver (UMLS:ICD10CM:K76.1); or
 Alcoholic cirrhosis of liver without ascites (UMLS:ICD10CM:K70.30); or
 Alcoholic cirrhosis of liver without ascites (UMLS:ICD10CM:K70.30); or
 Primary biliary cirrhosis (UMLS:ICD10CM:K74.3); or
 Alcoholic cirrhosis of liver with ascites (UMLS:ICD10CM:K70.31); or
 Hepatic fibrosis, unspecified (UMLS:ICD10CM:K74.00).

All the following must be satisfied:

 Group 1A: The terms in this group occurred at any time
 Patients must have:
 any of the following:
 Calculus of bile duct without cholangitis or cholecystitis without obstruction (UMLS:ICD10CM:K80.50); or
 Calculus of bile duct without cholangitis or cholecystitis (UMLS:ICD10CM:K80.5); or
 Cholangitis (UMLS:ICD10CM:K83.0); or
 Calculus of bile duct with cholecystitis (UMLS:ICD10CM:K80.4); or
 Calculus of bile duct without cholangitis or cholecystitis with obstruction (UMLS:ICD10CM:K80.51); or
 Calculus of bile duct with acute cholecystitis without obstruction (UMLS:ICD10CM:K80.42); or
 Calculus of bile duct with chronic cholecystitis without obstruction (UMLS:ICD10CM:K80.44); or
 Calculus of bile duct with cholecystitis, unspecified, without obstruction (UMLS:ICD10CM:K80.40); or
 Calculus of bile duct with acute cholecystitis with obstruction (UMLS:ICD10CM:K80.43); or
 Calculus of bile duct with chronic cholecystitis with obstruction (UMLS:ICD10CM:K80.45); or
 Calculus of bile duct with cholecystitis, unspecified, with obstruction (UMLS:ICD10CM:K80.41); or
 Calculus of gallbladder and bile duct without cholecystitis without obstruction (UMLS:ICD10CM:K80.70); or
 Calculus of gallbladder and bile duct without cholecystitis with obstruction (UMLS:ICD10CM:K80.71); or
 Calculus of gallbladder and bile duct with acute cholecystitis without obstruction (UMLS:ICD10CM:K80.62); or
 Calculus of gallbladder and bile duct with chronic cholecystitis without obstruction (UMLS:ICD10CM:K80.64); or
 Calculus of gallbladder and bile duct with acute cholecystitis with obstruction (UMLS:ICD10CM:K80.63); or
 Calculus of gallbladder and bile duct with acute and chronic cholecystitis without obstruction (UMLS:ICD10CM:K80.66); or
 Calculus of gallbladder and bile duct with acute and chronic cholecystitis with obstruction (UMLS:ICD10CM:K80.67); or
 Calculus of gallbladder and bile duct with chronic cholecystitis with obstruction (UMLS:ICD10CM:K80.65).
 Group 1B: Any instance of Group 1B occurred within 1 day before or up to 7 days after any instance of Group 1A
 Patients must have:
 all of the following:
 any of the following:
 Endoscopic retrograde cholangiopancreatography (ERCP); with removal of calculi/debris from biliary/pancreatic duct(s) (UMLS:CPT:43264); or
 Endoscopic retrograde cholangiopancreatography (ERCP); with placement of endoscopic stent into biliary or pancreatic duct, including pre- and post-dilation and guide wire passage, when performed, including sphincterotomy, when performed, each stent (UMLS:CPT:43274); or
 Endoscopic cannulation of papilla with direct visualization of pancreatic/common bile duct(s) (List separately in addition to code(s) for primary procedure) (UMLS:CPT:43273); or
 Endoscopic retrograde cholangiopancreatography (ERCP); diagnostic, including collection of specimen(s) by brushing or washing, when performed (separate procedure) (UMLS:CPT:43260); or
 Endoscopic retrograde cholangiopancreatography (ERCP); with biopsy, single or multiple (UMLS:CPT:43261); or
 Endoscopic retrograde cholangiopancreatography (ERCP); with sphincterotomy/papillotomy (UMLS:CPT:43262); or
 Endoscopic retrograde cholangiopancreatography (ERCP); with pressure measurement of sphincter of Oddi (UMLS:CPT:43263); or
 Endoscopic retrograde cholangiopancreatography (ERCP); with removal of calculi/debris from biliary/pancreatic duct(s) (UMLS:CPT:43264); or
 Endoscopic retrograde cholangiopancreatography (ERCP); with destruction of calculi, any method (eg, mechanical, electrohydraulic, lithotripsy) (UMLS:CPT:43265); or
 Endoscopic cannulation of papilla with direct visualization of pancreatic/common bile duct(s) (List separately in addition to code(s) for primary procedure) (UMLS:CPT:43273); or
 Endoscopic retrograde cholangiopancreatography (ERCP); with placement of endoscopic stent into biliary or pancreatic duct, including pre- and post-dilation and guide wire passage, when performed, including sphincterotomy, when performed, each stent (UMLS:CPT:43274); or
 Endoscopic retrograde cholangiopancreatography (ERCP); with removal of foreign body(s) or stent(s) from biliary/pancreatic duct(s) (UMLS:CPT:43275); or
 Endoscopic retrograde cholangiopancreatography (ERCP); with removal and exchange of stent(s), biliary or pancreatic duct, including pre- and post-dilation and guide wire passage, when performed, including sphincterotomy, when performed, each stent exchanged (UMLS:CPT:43276); or
 Endoscopic retrograde cholangiopancreatography (ERCP); with trans-endoscopic balloon dilation of biliary/pancreatic duct(s) or of ampulla (sphincteroplasty), including sphincterotomy, when performed, each duct (UMLS:CPT:43277); or
 Endoscopic retrograde cholangiopancreatography (ERCP); with ablation of tumor(s), polyp(s), or other lesion(s), including pre- and post-dilation and guide wire passage, when performed (UMLS:CPT:43278); and
 Platelets [#/volume] in Blood (TNX:9020) (at least 100.00 10*3/uL; and at least 18 years old at event).

# Appendix B – Text Representation of the Analysis Setup

This section contains the Index Event definition for each cohort.

The index event for Cohort 1 (query name: ERCP-Group A) is defined as the following:

Patients must have:
 any of the following:
 Unspecified cirrhosis of liver (UMLS:ICD10CM:K74.60); or
 Biliary cirrhosis, unspecified (UMLS:ICD10CM:K74.5); or
 Toxic liver disease with fibrosis and cirrhosis of liver (UMLS:ICD10CM:K71.7); or
 Primary biliary cirrhosis (UMLS:ICD10CM:K74.3); or
 Secondary biliary cirrhosis (UMLS:ICD10CM:K74.4); or
 Other and unspecified cirrhosis of liver (UMLS:ICD10CM:K74.6); or
 Alcoholic hepatitis with ascites (UMLS:ICD10CM:K70.11); or
 Chronic passive congestion of liver (UMLS:ICD10CM:K76.1); or
 Alcoholic cirrhosis of liver without ascites (UMLS:ICD10CM:K70.30); or
 Alcoholic cirrhosis of liver without ascites (UMLS:ICD10CM:K70.30); or
 Primary biliary cirrhosis (UMLS:ICD10CM:K74.3); or
 Alcoholic cirrhosis of liver with ascites (UMLS:ICD10CM:K70.31); or
 Hepatic fibrosis, unspecified (UMLS:ICD10CM:K74.00).

All the following must be satisfied:

 Group 1A: The terms in this group occurred at any time
 Patients must have:
 any of the following:
 Calculus of bile duct without cholangitis or cholecystitis without obstruction (UMLS:ICD10CM:K80.50); or
 Calculus of bile duct without cholangitis or cholecystitis (UMLS:ICD10CM:K80.5); or
 Cholangitis (UMLS:ICD10CM:K83.0); or
 Calculus of bile duct with cholecystitis (UMLS:ICD10CM:K80.4); or
 Calculus of bile duct without cholangitis or cholecystitis with obstruction (UMLS:ICD10CM:K80.51); or
 Calculus of bile duct with acute cholecystitis without obstruction (UMLS:ICD10CM:K80.42); or
 Calculus of bile duct with chronic cholecystitis without obstruction (UMLS:ICD10CM:K80.44); or
 Calculus of bile duct with cholecystitis, unspecified, without obstruction (UMLS:ICD10CM:K80.40); or
 Calculus of bile duct with acute cholecystitis with obstruction (UMLS:ICD10CM:K80.43); or
 Calculus of bile duct with chronic cholecystitis with obstruction (UMLS:ICD10CM:K80.45); or
 Calculus of bile duct with cholecystitis, unspecified, with obstruction (UMLS:ICD10CM:K80.41); or
 Calculus of gallbladder and bile duct without cholecystitis without obstruction (UMLS:ICD10CM:K80.70); or
 Calculus of gallbladder and bile duct without cholecystitis with obstruction (UMLS:ICD10CM:K80.71); or
 Calculus of gallbladder and bile duct with acute cholecystitis without obstruction (UMLS:ICD10CM:K80.62); or
 Calculus of gallbladder and bile duct with chronic cholecystitis without obstruction (UMLS:ICD10CM:K80.64); or
 Calculus of gallbladder and bile duct with acute cholecystitis with obstruction (UMLS:ICD10CM:K80.63); or
 Calculus of gallbladder and bile duct with acute and chronic cholecystitis without obstruction (UMLS:ICD10CM:K80.66); or
 Calculus of gallbladder and bile duct with acute and chronic cholecystitis with obstruction (UMLS:ICD10CM:K80.67); or
 Calculus of gallbladder and bile duct with chronic cholecystitis with obstruction (UMLS:ICD10CM:K80.65).
 Group 1B: Any instance of Group 1B occurred within 1 day before or up to 7 days after any instance of Group 1A
 Patients must have:
 all of the following:
 any of the following:
 Endoscopic retrograde cholangiopancreatography (ERCP); with removal of calculi/debris from biliary/pancreatic duct(s) (UMLS:CPT:43264); or
 Endoscopic retrograde cholangiopancreatography (ERCP); with placement of endoscopic stent into biliary or pancreatic duct, including pre- and post-dilation and guide wire passage, when performed, including sphincterotomy, when performed, each stent (UMLS:CPT:43274); or
 Endoscopic cannulation of papilla with direct visualization of pancreatic/common bile duct(s) (List separately in addition to code(s) for primary procedure) (UMLS:CPT:43273); or
 Endoscopic retrograde cholangiopancreatography (ERCP); diagnostic, including collection of specimen(s) by brushing or washing, when performed (separate procedure) (UMLS:CPT:43260); or
 Endoscopic retrograde cholangiopancreatography (ERCP); with biopsy, single or multiple (UMLS:CPT:43261); or
 Endoscopic retrograde cholangiopancreatography (ERCP); with sphincterotomy/papillotomy (UMLS:CPT:43262); or
 Endoscopic retrograde cholangiopancreatography (ERCP); with pressure measurement of sphincter of Oddi (UMLS:CPT:43263); or
 Endoscopic retrograde cholangiopancreatography (ERCP); with removal of calculi/debris from biliary/pancreatic duct(s) (UMLS:CPT:43264); or
 Endoscopic retrograde cholangiopancreatography (ERCP); with destruction of calculi, any method (eg, mechanical, electrohydraulic, lithotripsy) (UMLS:CPT:43265); or
 Endoscopic cannulation of papilla with direct visualization of pancreatic/common bile duct(s) (List separately in addition to code(s) for primary procedure) (UMLS:CPT:43273); or
 Endoscopic retrograde cholangiopancreatography (ERCP); with placement of endoscopic stent into biliary or pancreatic duct, including pre- and post-dilation and guide wire passage, when performed, including sphincterotomy, when performed, each stent (UMLS:CPT:43274); or
 Endoscopic retrograde cholangiopancreatography (ERCP); with removal of foreign body(s) or stent(s) from biliary/pancreatic duct(s) (UMLS:CPT:43275); or
 Endoscopic retrograde cholangiopancreatography (ERCP); with removal and exchange of stent(s), biliary or pancreatic duct, including pre- and post-dilation and guide wire passage, when performed, including sphincterotomy, when performed, each stent exchanged (UMLS:CPT:43276); or
 Endoscopic retrograde cholangiopancreatography (ERCP); with trans-endoscopic balloon dilation of biliary/pancreatic duct(s) or of ampulla (sphincteroplasty), including sphincterotomy, when performed, each duct (UMLS:CPT:43277); or
 Endoscopic retrograde cholangiopancreatography (ERCP); with ablation of tumor(s), polyp(s), or other lesion(s), including pre- and post-dilation and guide wire passage, when performed (UMLS:CPT:43278); and
 Platelets [#/volume] in Blood (TNX:9020) (between 30.00 and 100.00 10*3/uL; and at least 18 years old at event).

The index event for Cohort 2 (query name: ERCP-Group B) is defined as the following:

All the following must be satisfied:

 Group 1A: The terms in this group occurred at any time
 Patients must have:
 any of the following:
 Calculus of bile duct without cholangitis or cholecystitis without obstruction (UMLS:ICD10CM:K80.50); or
 Calculus of bile duct without cholangitis or cholecystitis (UMLS:ICD10CM:K80.5); or
 Cholangitis (UMLS:ICD10CM:K83.0); or
 Calculus of bile duct with cholecystitis (UMLS:ICD10CM:K80.4); or
 Calculus of bile duct without cholangitis or cholecystitis with obstruction (UMLS:ICD10CM:K80.51); or
 Calculus of bile duct with acute cholecystitis without obstruction (UMLS:ICD10CM:K80.42); or
 Calculus of bile duct with chronic cholecystitis without obstruction (UMLS:ICD10CM:K80.44); or
 Calculus of bile duct with cholecystitis, unspecified, without obstruction (UMLS:ICD10CM:K80.40); or
 Calculus of bile duct with acute cholecystitis with obstruction (UMLS:ICD10CM:K80.43); or
 Calculus of bile duct with chronic cholecystitis with obstruction (UMLS:ICD10CM:K80.45); or
 Calculus of bile duct with cholecystitis, unspecified, with obstruction (UMLS:ICD10CM:K80.41); or
 Calculus of gallbladder and bile duct without cholecystitis without obstruction (UMLS:ICD10CM:K80.70); or
 Calculus of gallbladder and bile duct without cholecystitis with obstruction (UMLS:ICD10CM:K80.71); or
 Calculus of gallbladder and bile duct with acute cholecystitis without obstruction (UMLS:ICD10CM:K80.62); or
 Calculus of gallbladder and bile duct with chronic cholecystitis without obstruction (UMLS:ICD10CM:K80.64); or
 Calculus of gallbladder and bile duct with acute cholecystitis with obstruction (UMLS:ICD10CM:K80.63); or
 Calculus of gallbladder and bile duct with acute and chronic cholecystitis without obstruction (UMLS:ICD10CM:K80.66); or
 Calculus of gallbladder and bile duct with acute and chronic cholecystitis with obstruction (UMLS:ICD10CM:K80.67); or
 Calculus of gallbladder and bile duct with chronic cholecystitis with obstruction (UMLS:ICD10CM:K80.65).
 Group 1B: Any instance of Group 1B occurred within 1 day before or up to 7 days after any instance of Group 1A
 Patients must have:
 all of the following:
 any of the following:
 Endoscopic retrograde cholangiopancreatography (ERCP); with removal of calculi/debris from biliary/pancreatic duct(s) (UMLS:CPT:43264); or
 Endoscopic retrograde cholangiopancreatography (ERCP); with placement of endoscopic stent into biliary or pancreatic duct, including pre- and post-dilation and guide wire passage, when performed, including sphincterotomy, when performed, each stent (UMLS:CPT:43274); or
 Endoscopic cannulation of papilla with direct visualization of pancreatic/common bile duct(s) (List separately in addition to code(s) for primary procedure) (UMLS:CPT:43273); or
 Endoscopic retrograde cholangiopancreatography (ERCP); diagnostic, including collection of specimen(s) by brushing or washing, when performed (separate procedure) (UMLS:CPT:43260); or
 Endoscopic retrograde cholangiopancreatography (ERCP); with biopsy, single or multiple (UMLS:CPT:43261); or
 Endoscopic retrograde cholangiopancreatography (ERCP); with sphincterotomy/papillotomy (UMLS:CPT:43262); or
 Endoscopic retrograde cholangiopancreatography (ERCP); with pressure measurement of sphincter of Oddi (UMLS:CPT:43263); or
 Endoscopic retrograde cholangiopancreatography (ERCP); with removal of calculi/debris from biliary/pancreatic duct(s) (UMLS:CPT:43264); or
 Endoscopic retrograde cholangiopancreatography (ERCP); with destruction of calculi, any method (eg, mechanical, electrohydraulic, lithotripsy) (UMLS:CPT:43265); or
 Endoscopic cannulation of papilla with direct visualization of pancreatic/common bile duct(s) (List separately in addition to code(s) for primary procedure) (UMLS:CPT:43273); or
 Endoscopic retrograde cholangiopancreatography (ERCP); with placement of endoscopic stent into biliary or pancreatic duct, including pre- and post-dilation and guide wire passage, when performed, including sphincterotomy, when performed, each stent (UMLS:CPT:43274); or
 Endoscopic retrograde cholangiopancreatography (ERCP); with removal of foreign body(s) or stent(s) from biliary/pancreatic duct(s) (UMLS:CPT:43275); or
 Endoscopic retrograde cholangiopancreatography (ERCP); with removal and exchange of stent(s), biliary or pancreatic duct, including pre- and post-dilation and guide wire passage, when performed, including sphincterotomy, when performed, each stent exchanged (UMLS:CPT:43276); or
 Endoscopic retrograde cholangiopancreatography (ERCP); with trans-endoscopic balloon dilation of biliary/pancreatic duct(s) or of ampulla (sphincteroplasty), including sphincterotomy, when performed, each duct (UMLS:CPT:43277); or
 Endoscopic retrograde cholangiopancreatography (ERCP); with ablation of tumor(s), polyp(s), or other lesion(s), including pre- and post-dilation and guide wire passage, when performed (UMLS:CPT:43278); and
 Platelets [#/volume] in Blood (TNX:9020) (at least 100.00 10*3/uL; and at least 18 years old at event).

# Appendix C – Text Representation of the Outcomes Definition

This analysis includes the following outcomes:

Sepsis
 Patients must have:
 Other sepsis (UMLS:ICD10CM:A41).

AKI
 Patients must have:
 any of the following:
 Acute kidney failure (UMLS:ICD10CM:N17); or
 Acute kidney failure with tubular necrosis (UMLS:ICD10CM:N17.0); or
 Acute kidney failure, unspecified (UMLS:ICD10CM:N17.9); or
 Other acute kidney failure (UMLS:ICD10CM:N17.8); or
 Acute kidney failure with acute cortical necrosis (UMLS:ICD10CM:N17.1); or
 Acute kidney failure with medullary necrosis (UMLS:ICD10CM:N17.2).

SBP
 Patients must have:
 Spontaneous bacterial peritonitis (UMLS:ICD10CM:K65.2).

All-cause mortality
 Patients must have:
 any of the following:
 Ill-defined and unknown cause of mortality (UMLS:ICD10CM:R99); or
 Ill-defined and unknown cause of mortality (R99) (UMLS:ICD10CM:R99-R99); or
 Deceased (Deceased).

Jaundice
 Patients must have:
 Unspecified jaundice (UMLS:ICD10CM:R17).

Septic shock
 Patients must have:
 any of the following:
 Severe sepsis with septic shock (UMLS:ICD10CM:R65.21); or
 Other sepsis (UMLS:ICD10CM:A41); or
 Postprocedural septic shock (UMLS:ICD10CM:T81.12); or
 Postprocedural septic shock, initial encounter (UMLS:ICD10CM:T81.12XA); or
 Postprocedural septic shock, sequela (UMLS:ICD10CM:T81.12XS); or
 Postprocedural septic shock, subsequent encounter (UMLS:ICD10CM:T81.12XD); or
 Sepsis due to Methicillin susceptible Staphylococcus aureus (UMLS:ICD10CM:A41.01); or
 Streptococcal sepsis (UMLS:ICD10CM:A40).

Post-ERCP bleeding
 Patients must have:
 any of the following:
 Postprocedural hemorrhage of a digestive system organ or structure following a digestive system procedure (UMLS:ICD10CM:K91.840); or
 Hemorrhage, not elsewhere classified (UMLS:ICD10CM:R58).

Post-ERCP Pancreatitis
 Patients must have:
 any of the following:
 Biliary acute pancreatitis (UMLS:ICD10CM:K85.1); or
 Biliary acute pancreatitis with uninfected necrosis (UMLS:ICD10CM:K85.11); or
 Biliary acute pancreatitis with infected necrosis (UMLS:ICD10CM:K85.12).

Need for blood products transfusion
 Patients must have:
 Transfusion, blood or blood components (UMLS:CPT:36430).
